# Coupled Gel Coprecipitation and Hydrothermal Processing to Synthesise Cubic Structured Compounds in the SrTiO_3_–SrZrO_3_ System

**DOI:** 10.3390/gels12060505

**Published:** 2026-06-06

**Authors:** Juan Carlos Rendón-Angeles, Zully Matamoros-Veloza, Diego Emiliano Carrillo-Ramírez, José Remigio Quiñones-Gurrola, Kazumichi Yanagisawa

**Affiliations:** 1 Centre for Research and Advanced Studies of National Polytechnic Institute, Saltillo-Campus, Ramos Arizpe 25900, Mexico; diego.carrillo@cinvestav.mx (D.E.C.-R.);; 2 Graduate Division, Technological Institute of Saltillo, Tecnológico Nacional de México, Saltillo 25280, Mexico; 3 Research Laboratory of Hydrothermal Chemistry, Faculty of Science, Kochi University, Kochi 780-8073, Japan

**Keywords:** gel coprecipitation, hydrothermal synthesis, solid solutions, cubic structure, orthorhombic structure

## Abstract

The sol–gel coprecipitation method is highly efficient for synthesising a wide range of binary perovskite solid solutions (SSs), which have been under exhaustive study due to their semiconductor and catalytic properties. Therefore, we conducted a systematic study to extend the chemical stability of the cubic structure in Zr^4+^-rich SS in the system SrTiO_3_–SrZrO_3_. The proposed new approach involves in situ gel coprecipitation and simultaneous hydrothermal processing, which was conducted at standard conditions (200 °C for 6 h) in a KOH (5 M) solution under stirring at 130 rpm. The formation of the cubic perovskite-structured SS occurred in the compositional range from 10.0 to 100.0 mol% Ti^4+^. The particle crystallisation was achieved via the dissolution-crystallisation mechanism, which proceeded rapidly, aided by preliminary gel dehydration and vigorous stirring. The prepared particles, either orthorhombic or cubic, have a unique morphology, resembling a pseudocuboidal shape with rounded edges. The particle size decreases as the Ti^4+^ content in the SSs increases, due to improved gel solubility. The band gap of the cubic intermediate SSs is sharp, ranging from 3.12 to 3.57 eV; thus, these perovskites can be applied in the development of semiconductor devices and in catalysis. These powders can also be employed as cool pigments due to their high NIR solar irradiance of 80.22%.

## 1. Introduction

The development of perovskite-structured oxide solid solutions (SSs) has recently been the subject of research by various groups worldwide. The efforts to explore specific compositions, namely the binary system SrTiO_3_–SrZrO_3_, were driven by the continuous synergy of material innovation, necessitated by the need for efficient information storage, energy storage devices, spintronics, oxygen sensors, and other applications [1,2,3,4]. Furthermore, the SrTiO_3_–SrZrO_3_ system is technologically important due to its dielectric properties [5], and research interest in the preparation of SSs at the B-site cation isomorphous substitution has recently increased. Additionally, new efforts have been devoted to exploring alternative processing approaches to optimise the chemical and physical properties of the binary system end-member phases. Among the strontium-based perovskite oxides, SrZrO_3_ exhibits the highest bandgap value, 5.6 eV, which provides either high negative or positive reduction potentials applied to catalytic reactions, in comparison with other perovskites [5]. A pioneering study was conducted to prepare orthorhombic-structured Zr-rich solid solutions (SrZr_1−x_Ti_x_O_3_), with Ti^4+^ content ranging from 0.0 to 25 mol%. The photocatalytic hydrogen production capability and band gap measurements of these materials were investigated. Remarkably, the increase of Ti^4+^ content up to 20.0 mol% narrowed the bandgap and boosted hydrogen production from ethanol degradation by a factor of 35 [5]. Thus, the tuning of SrZr_0.8_Ti_0.2_O_3_ photocatalytic performance results from combining at least two elements that maintain cationic structural ordering, thereby enabling the development of advanced new perovskite materials [6,7].

Hitherto, pioneering work on preparing SS-SrTi_1−x_Zr_x_O_3_ has been achieved through systematic studies of Zr^4+^ doping, specifically in the compositional range of 0.0 ≤ x ≤ 0.5 mol% Zr^4+^, by using the conventional high-temperature solid-state reaction [8,9,10,11,12,13]. SS formation is kinetically driven by the chemical reactivity of the powder mixture, composed of high-purity SrCO_3_, TiO_2,_ and ZrO_2_ powders. The homogeneously distributed powder mixture is pelletized and subsequently calcined at temperatures between 800 and 1400 °C for various reaction times (24–96 h). However, the synthesis of highly monodisperse SS-SrTi_1−x_Zr_x_O_3_ nanoparticles using this technique has not yet been conducted, and the product’s chemical compositional homogeneity is also compromised; these factors are among the main disadvantages of this ceramic processing route.

In contrast, the synthesis of nanostructured SS-perovskites via chemical solution processing has been only partially explored at temperatures below 800 °C. Recently, several pioneering investigations using sol–gel [5,14] and coprecipitation [15] techniques have demonstrated their effectiveness in preparing nanoparticles of SS-SrTi_1−x_Zr_x_O_3_. The powders exhibit a monomodal particle size distribution and homogeneous stoichiometric compositions, which optimise their physical and chemical properties [5,14,15]. A different approach involved reacting a pasty hydrous Ti-gel (Ti(OH)_4_•4.5H_2_O) with the SrSO_4_ mineral precursor under hydrothermal conditions at 250 °C for 24 h in a 5 M KOH solution [16]. Under these conditions, the dissolution of SrSO_4_, coupled with the dehydration of the Ti-gel, established the chemical equilibrium required to trigger the crystallisation of micron-sized, cubic-shaped SrTiO_3_ crystals. This process is analogous to soft chemical methods driven by a topochemical reaction mechanism, resulting from the reaction of the colloidal gel with an alkaline medium bearing Sr^2+^ ions [17,18,19]. Likewise, a pioneering attempt employed precipitated pasty gels Ti(OH)_4_•4.5H_2_O and Zr(OH)_4_•9.64H_2_O to prepare SrTi_1−x_Zr_x_O_3_ and SrZr_1−x_Ti_x_O_3_ solid solutions under hydrothermal conditions. The treatment was conducted using SrSO_4_ powder and a mixture of both Ti and Zr gels; these precursors were dispersed in the alkaline fluid by constant stirring at 130 rpm. Particles with an orthorhombic structure corresponding to Zr-rich SSs were produced with Ti^4+^ contents up to 7.5 mol% (SrZr_0.925_Ti_0.075_O_3_). On the contrary, in the range between 7.5 and 47.5 mol% Ti^4+^, the preferential crystallisation of two Zr-rich powders composed of varied amounts of Ti^4+^ with orthorhombic and cubic structures was observed. The powders obtained at high Ti^4+^ concentrations (47.5–100.0 mol%) exhibited a cubic structure. These results differ from those obtained by solid-state reaction, because the tetragonal perovskite phase is stable in the intermediate compositional range of 40.0–90.0 mol% Ti^4+^ [9,11]. Consensus over the stability of the perovskite cubic crystal structure is crucial for preparing chemically stable SrZr_1−x_Ti_x_O_3_ or SrTi_1−x_Zr_x_O_3_ SSs; thus, alternative approaches based on low-temperature chemical processing must be investigated to develop processing methods for synthesising perovskite solid solutions with microstructural control (particle size and morphology).

According to a survey of the literature, none of the numerous research studies carried out to synthesise powders of perovskite compounds in the binary SrTiO_3_–SrZrO_3_ system have employed alkaline coprecipitated gel, even under alkaline hydrothermal conditions. The present work is focused on investigating the feasibility of using coprecipitated SrTi_1−x_Zr_x_(OH)_6_•nH_2_O and SrZr_1−x_Ti_x_(OH)_6_•nH_2_O gel precursors as a relatively rapidly dissolving source (Sr^2+^, Ti^4+^ and Zr^4+^) during the crystallisation of SrTi_1−x_Zr_x_O_3_ or SrZr_1−x_Ti_x_O_3_ solid solutions with cubic and orthorhombic crystalline structures. Attempts were made to evaluate the reactivity of coprecipitated gels with different molar ratios of Ti: Zr (0.0–100.0 molar% Ti^4+^) in a highly concentrated KOH fluid (5 M) at low temperature (200 °C) and to determine the potential of the soft chemical processing approach investigated at lower temperatures (125–175 °C), which was only applied for the intermediate SrTi_0.5_Zr_0.5_O_3_ solid solution. Hypothetically, the coprecipitated gel might dissolve rapidly in alkaline media, triggering the rapid solvent-fluid supersaturation with the solute, thereby causing the spontaneous crystallisation of powders with specific chemical compositions and cubic or orthorhombic structures. A particular emphasis was intended to determine the stability of the perovskite cubic structure in the compositional range of 10.0 ≤ x ≤ 100.0 mol% of Ti^4+^ for the SrTi_1−x_Zr_x_O_3_ and SrZr_1−x_Ti_x_O_3_ (SS) samples. Additionally, microstructural analyses were conducted to determine morphology, particle size and optical properties (reflectance and band gap energy). Potential applications of the investigated perovskite compounds were proposed based on the optical properties.

## 2. Results and Discussion

### 2.1. Synthesis of Single-Phase SrZr_1−x_Ti_x_O_3_ and SrTi_1−x_Zr_x_O_3_ SSs Using One-Pot Chemical Processing

One critical factor that triggers the simultaneous formation of two perovskite SSs under hydrothermal conditions is the precursor sol–gel dehydration; this reaction proceeds more slowly on tetravalent hydrous phases, such as Ti(OH)_4_•4.5H_2_O and Zr(OH)_4_•9.64H_2_O [20]. Consequently, the dissolution of the dehydrated gel is rather slow or fast depending on the gel’s chemical stability; this reaction trend provokes a variation in the bulk molar solute supersaturation (Zr^4+^ and Ti^4+^) molar volume, which causes a compositional fluctuation during crystallisation, provoking the simultaneous formation of two perovskite orthorhombic and cubic structured compounds. This process occurred when the precipitated gels were used to crystallise Zr-rich SSs (SrZr_1−x_Ti_x_O_3_) over the range of 10.0 ≤ x ≤ 47.5 mol% Ti^4+^. In contrast, a coprecipitated gel composed of three cations in specific stoichiometric ratios might react rapidly, resulting in limited differences in the molar content of the solute during the reaction [20]. Indeed, naked-eye inspection revealed that no traces of the parent gel remained, and the powder product was white despite the change in the SS composition. The results of the relevant experiments aimed at investigating the feasibility of producing single-phase perovskite SSs are summarised in Table 1, which includes details of the crystalline structure of each SS prepared. These results reveal that the proposed approach effectively overcomes the processing disadvantage associated with gel dissolution. It is worth emphasising that the coprecipitated gel enhanced the crystallisation of a series of perovskite compounds with cubic (10.0–100.0 mol% Ti^4+^) and orthorhombic (0.0–10.0 mol% Ti^4+^) structures at low temperature for 6 h under hydrothermal conditions.

### 2.2. Crystalline Structural Features of the Hydrothermally Synthesised SrZr_1−x_Ti_x_O_3_ and SrTi_1−x_Zr_x_O_3_ SSs

**Figure 1:**
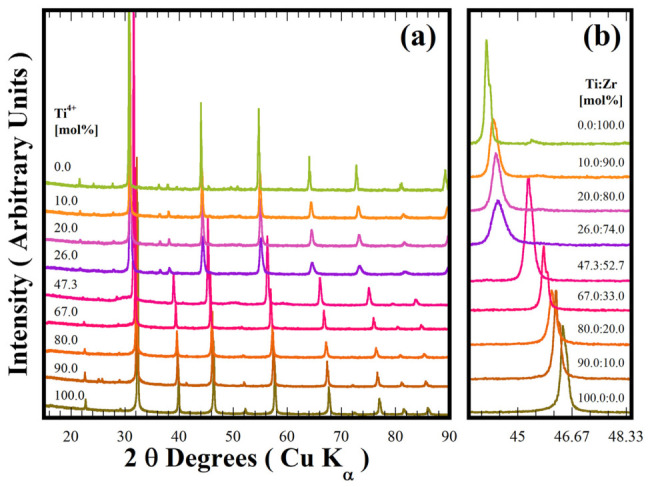
(**a**,**b**) X–ray diffraction patterns of the solid solutions of SrTi_1−x_Zr_x_O_3_ and SrZr_1−x_Ti_x_O_3_ obtained hydrothermally with 5 M KOH at 200 °C for 6 h.

The typical powder diffraction patterns of the reaction products prepared under standard hydrothermal conditions at 200 °C for 6 h with constant stirring (130 rpm) and varying Ti^4+^ content are shown in Figure 1. In general, naked-eye observation revealed that the coprecipitated colloid reacted with the alkaline liquor, resulting in the crystallisation of a fine white powder in all the experiments described in Table 1. When the coprecipitated SrTi(OH)_6_•4.5H_2_O and SrZr(OH)_6_•9.64H_2_O gels were hydrothermally treated separately, the peaks in the PXRD patterns were indexed with those corresponding to the single-phase cubic SrTiO_3_ (space group *Pm3m*, ICDD card No. 40-1500) and SrZrO_3_ orthorhombic (ICDD 70-0283 space group *Pbnm*) perovskite structures, respectively. Interestingly, at Ti^4+^ contents over 10.0 mol%, all the peaks of the cubic structure in the diffraction pattern within the 2θ angle range between 22.0 and 90.0° exhibited a progressive shift to lower 2θ angles, as seen in Figure 1a. This peak displacement occurred gradually as the Ti^4+^ content increased from 10.0 to 100.0 mol%. Thus, these results indicate that both SrZr_1−x_Ti_x_O_3_ and SrTi_1−x_Zr_x_O_3_ SSs (SS1) with a cubic structure are highly stable in the interval of 10.0 mol% ≤ x ≤ 100.0 mol% Ti^4+^. Moreover, perovskite compounds (SrTi_1−x_Zr_x_O_3_) with a tetragonal structure have been produced at mild temperatures below 120 °C [21] and at high temperatures above 1200 °C [9,11,22]. To confirm the existence of the tetragonal structure on the hydrothermally produced powders, a detailed analysis of the PXRD patterns was carried out in the 2θ range from 44° to 48°, with particular attention to the peak at 46° (Figure 1b). The shape of the peak observed in our samples differs from the typical doublet peak associated with the tetragonal structure, ruling out the presence of the tetragonal structure in the reaction product SS1. The two overlapping peaks correspond to the crystallographic diffraction planes (002) and (200) of the tetragonal structure (space group I4/*mmm*) [21,23,24]. Likewise, the peaks of the orthorhombic perovskite structure were not detected in the Zr-rich SSs (SrZr_1−x_Ti_x_O_3_) prepared in the range of 10.0 mol% to 50.0 mol% Ti^4+^. It is worth emphasising that the orthorhombic triplet peak at 55.5° was only revealed in the SrZrO_3_ sample. The characteristic peak differs in shape from those on the synthesised SrZr_1−x_Ti_x_O_3_ SSs, which are symmetric and shift slightly towards larger angles. The crystalline structural analyses confirmed that the Zr^4+-^ rich SSs (SrZr_1−x_Ti_x_O_3_) belong to the cubic perovskite structure with space group *Pm3m*. We argue that the SS synthesis under hydrothermal conditions is controlled by the coprecipitated gel’s dissolution rate, which varies due to its nominal composition. The higher the Ti^4+^ concentration in the gel (SrZr_1−x_Ti_x_(OH)_6_•nH_2_O and SrTi_1−x_Zr_x_(OH)_6_•nH_2_O gels; Equations (S4) and (S7) in Supplementary supporting information file), the more rapidly it dissolves in the alkaline solution. Consequently, the solvent supersaturation of the solute rapidly enhances the crystallisation of single-phase, cubic or Zr-rich orthorhombic (SrZr_1−x_Ti_x_O_3_ in the range of 90.0–100.0 mol% Zr^4+^, named SS2) particles. This inference is supported by the gel’s dissolution differences enhanced under analogous hydrothermal conditions previously reported [20]. The gel mixture consisting of precipitated Ti(OH)_4_•4.5H_2_O and Zr(OH)_4_•9.64H_2_O provoked the simultaneous crystallisation of powders with orthorhombic and cubic structures with Zr^4+^ contents between 47.5 and 92.5 mol%.

**Figure 2:**
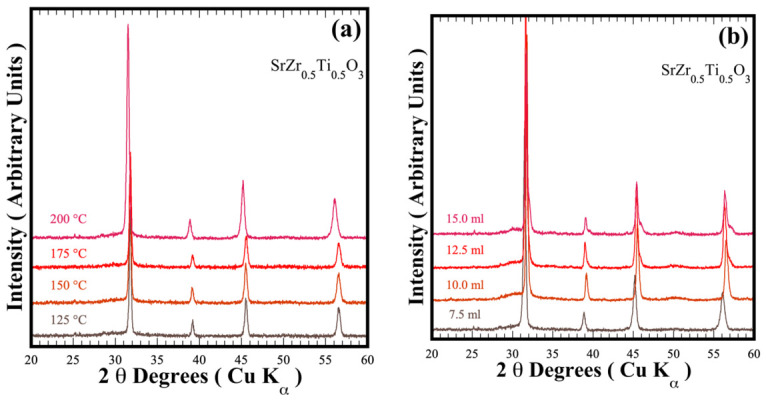
SrTi_0.5_Zr_0.5_O_3_ powders were synthesised at standard conditions with 5 M KOH for 6 h with 7.5 mL of the precursor solutions at (**a**) various temperatures and at 200 °C for 6 h with (**b**) different cation solution volumes.

On the other hand, the chemical stability of the cubic-structured SrTi_0.5_Zr_0.5_O_3_ SS was investigated at various reaction temperatures. Likewise, experiments were conducted with various volumes of metal salt solutions, and the 5 M NaOH solution was also varied. Generally, the XRD patterns of the powders prepared at various temperatures (125–200 °C) for 6 h exhibited only the characteristic peaks of the cubic-structured perovskite (ICDD card No. 40-1500). The PXRD patterns of the powders prepared below 175 °C displayed peaks at nearly the same 2θ angle positions (Figure 2a), indicating that chemical composition does not diverge from the nominal SrTi_0.5_Zr_0.5_O_3_. Although the XRD pattern of the powder prepared at 200 °C shifted slightly towards lower diffraction angles, this behaviour is irrespective of either the reaction temperature or the residual thermal strain; thus, a slight compositional variation is likely to further enhance this effect. Indeed, the element content calculated by energy-dispersive X-ray spectrometry using an X-ray microprobe indicated that the average contents of Ti^4+^ and Zr^4+^ were 47.3 mol% and 52.7 mol%, respectively (±1.5 mol%). This result is consistent with the gradual shift in PXRD pattern observed for other SS SrZr_1−x_Ti_x_O_3_ powders prepared with high Zr^4+^ contents. Moreover, a similar reaction trend was observed for samples prepared with different volumes of the metal salt solutions. The composition of the SSs prepared at volumes above 10 mL is nearly similar to the nominal selected SrTi_0.5_Zr_0.5_O_3_. The average contents of Ti and Zr determined by EDX analyses were 50.1 ± 1.0 mol% and 49.9 ± 1.0 mol%, respectively. However, the sample prepared at the standard conditions had a chemical composition of 47.3 mol% Ti^4+^ and 52.7 mol% Zr^4+^. The chemical composition of the selected powder, as measured by EDX, is shown in Table S3 of the SSIF. In addition, these results reveal that the coprecipitated gel is highly reactive in alkaline media, even when the net KOH concentration in the reaction media is markedly diluted from 3.25 M (55 mL) to 1.47 M (25 mL), as shown in Table 1. Based on these data, we assume that both cubic (SS1) and orthorhombic (SS2) phases can be prepared via a single-step reaction, triggered by coprecipitated gel steady-state dissolution, thereby allowing the solute saturation with the desired stoichiometric ratio [23]. Consequently, the SS particle crystallisation occurs rapidly even under hydrothermal conditions at 125 °C, with a net KOH alkalinity of 1.47 M.

**Figure 3:**
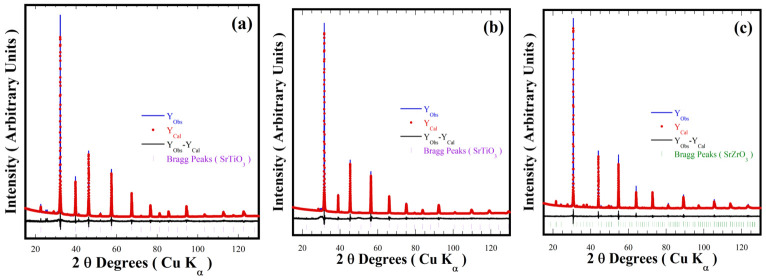
Rietveld refinement plots of products obtained hydrothermally using coprecipitated gels prepared with different cation molar Ti^4+^:Zr^4+^ ratios of (**a**) 0.67:0.33, (**b**) 0.26:0.74 and (**c**) 0.0:100.0 mol%, with a solvent (5 M KOH) volume filling ratio of 20% at 200 °C for 6 h.

The crystalline aspects of both the SS1 cubic and SS2 orthorhombic powders, such as the lattice parameters, lattice strain, and crystallite size, were systematically investigated by Rietveld refinement analyses, which included fitting parameters for background, thermal isotropy, scale factor, profile half-width, atomic fraction occupation and the physical parameters of the XRD machine goniometer. In addition, a subroutine to verify the presence of a tetragonal structure was also included in the refinement algorithm. The typical plots of selected samples prepared with different Ti^4+^ and Zr^4+^ contents are shown in Figure 3. It is worth emphasising that the residual line between the experimental and calculated XRD data exhibits a linear trend; similar results were obtained for all samples prepared under the experimental conditions described in Table 1. Therefore, the proposed refinement algorithm provided adequate accuracy in calculating the main crystalline features of both the SS1 cubic and SS2 orthorhombic products, selected from the entire binary SrTiO_3_–SrZrO_3_ system. Moreover, the precision of the refinement approach is supported by low average values of the goodness-of-fit factor GOF (χ^2^ = 4.3 ± 1.4), *R_wp_* (5.49 ± 2.4) and other fitting factors such as *R_p_*, *R_exp_* and *R_Bragg_*; all data are shown in Table 1. Indeed, the refinement plots in Figure 3a,b of the intermediate perovskite-structured SS1s revealed a straight residual line in all cases, which resembles the algorithm’s adequate accuracy for fitting the experimental and calculated XRD patterns. The calculated molar concentration of tetravalent elements was included in the atomic occupation algorithm subroutine, resulting in a marked reduction in fitting parameter values. Likewise, the existence of particles with a tetragonal structure was neglected because the refining algorithm failed to fit this structure in the experimental pattern. Therefore, based on these crystalline results, the formation of cubic-structured SrTi_0.67_Zr_0.33_O_3_ and SrZr_0.74_Ti_0.26_O_3_ solid solutions via in situ gel coprecipitation coupled with hydrothermal processing was confirmed. Furthermore, typical Rietveld plots of the residual cubic SS1s, including the SrTiO_3_ sample, are shown in Figure S1 of the Supplementary supporting information file.

**Figure 4:**
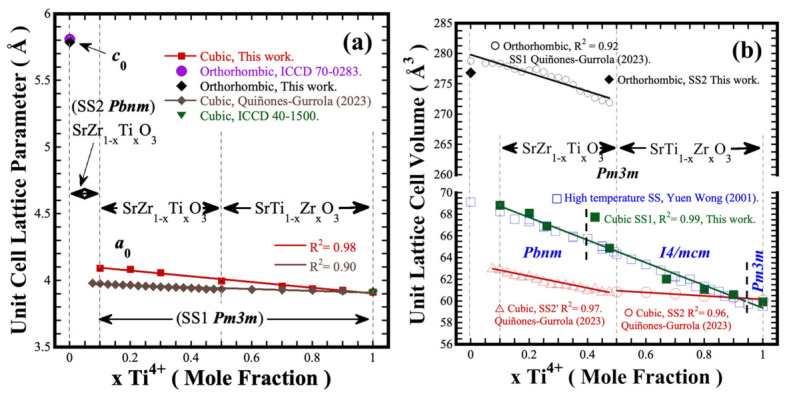
Crystalline structural parameter variation of perovskite structure of cubic SS1 (◼, ◼, SrTi_1−x_Zr_x_O_3_ or SrZr_1−x_Ti_x_O_3_) and orthorhombic SS2 (⯁, SrZr_1−x_Ti_x_O_3_), prepared under hydrothermal conditions at 200 C for 6 h with stirring at 130 rpm. (**a**) Unit cell lattice parameters and (**b**) unit cell volume. Unit cell values of single phases (▼) SrTiO_3_ Tausonite and (⬤) SrZrO_3_ and those of the SrTi_1−x_Zr_x_O_3_ solid solutions produced at (☐) a high temperature [11] and (⯁) hydrothermally [20] within the entire compositional range of the binary system SrZrO_3_–SrTiO_3_.

On the other hand, the calculated unit-cell lattice parameters of the compounds prepared hydrothermally under standard conditions (200 °C for 6 h, 5 M KOH), as a function of the Ti^4+^ content, are plotted in Figure 4a. This plot also includes, for comparison purposes, the “*a*_0_” values of the cubic-structured SSs (brown line) hydrothermally prepared using mixtures of the Ti^4+^ and Zr^4+^ pasty gels prepared separately. Generally, the results revealed that the calculated lattice parameters of the single-phase SrTiO_3_ (◼) and SrZrO_3_ (⯁) were nearly similar to those reported in the ICCD cards Nos. 40-1500 (▼) and 70-0283 (⬤), respectively. Furthermore, the lattice parameter “*a*_0_*”* of the intermediate cubic perovskite structured SS1s exhibited a gradual increase when Ti^4+^ contents below 90.0 mol% were added to coprecipitate the gels prior to the hydrothermal treatment. Interestingly, the “*a*_0_*”* values had a dispersion coefficient (R^2^) of 0.98; thus, these data are consistent with the systematic peak displacement caused by the incorporation of Zr^4+^ atoms, which have a larger atomic radius (0.16 nm) than that of Ti^4+^ (0.144 nm). This phenomenon agrees with the feature revealed in the XRD patterns shown in Figure 1. Therefore, this trend indicates the dependence of Zr^4+^ incorporation in the one-step synthesis of the B-site-substituted perovskite powders under hydrothermal conditions. Likewise, the linear trend of the cubic-structured SS1s agrees with Vegard’s law; therefore, the process investigated is likely optimum for preparing thermodynamically stable rich SrTi_1−x_Zr_x_O_3_ or SrZr_1−x_Ti_x_O_3_ within a broad Ti^4+^ concentration (10.0 ≤ x ≤ 100.0 mol% Ti^4+^). Indeed, the lattice parameters of SS1 powders produced in this work are slightly larger than those of SS2’ prepared under similar hydrothermal conditions under stirring (130 rpm) within the Ti^4+^ range of 10.0–50.0 mol% [20]. The differences in lattice constants are likely due to residual strain induced structurally during the crystallisation stage, occurring even at low temperature (200 °C) [25]. The level of residual strain is markedly affected by reaction conditions that enhance the particle synthesis. In our case, the coprecipitated gel’s chemical reactivity accelerates the reactions that induce and enhance the particle crystallisation process under the hydrothermal conditions explored.

Interestingly, the lattice unit-cell volumes of both perovskite SS1 (cubic) and SS2 (orthorhombic) exhibited a similar variation of their lattice constants “*a*_0_” as seen in Figure 4b. The unit cell volume of the SSs SrZr_1−x_Ti_x_O_3_ (☐) prepared by solid-state reaction at 1400 °C for 96 h [11] and those of the SS2’ (△) and SS2 (◯), hydrothermally prepared [20], were included for comparison purposes. Generally, the lattice volumes of the cubic SS1s (◼) exhibited a linear dependence with a narrow dispersion coefficient (R^2^) of 0.99, which depends on the Ti^4+^ content incorporated in the perovskite structure B site. In the context of the chemical composition for the binary SrTiO_3_–SrZrO_3_ system, the linear variation in the lattice volume corroborates that a series of solid solutions with cubic perovskite can be produced in the range of 10.0–100.0 mol% Ti^4+^ by the coupled gel coprecipitation-hydrothermal process. However, the lattice cell volumes of the hydrothermally prepared SS1 powders are seemingly consistent with the linear trend observed in analogous SSs prepared in the entire binary SrTiO_3_–SrZrO_3_ system [9,11]. The formation of SSs with three perovskite structures proceeded at high temperature (1400 °C) by solid-state reaction. Interestingly, the tetragonal structure is the major predominant phase produced in a wider compositional range, namely 40.0–95.0 mol% Ti^4+^, in comparison with the orthorhombic (0.0–40.0 mol% Ti^4^) and cubic (95.0–100.0 mol% Ti^4+^) structured SSs. The formation of each structure was achieved through long-range atomic ordering of the octahedral BO_6_ units caused by the heat treatment. These structural features distort the basic lattice symmetry, leading to specific crystallographic superlattice reflections normally detected by neutron diffraction or XRD [9,11]. However, the typical superlattice reflections at 2θ = 33° and 41° (021 and 122/212 Miller indexes) and at 2θ ≈ 40° (121) that provide the orthorhombic and tetragonal structure stability were not detected in the diffraction patterns of the SS1s hydrothermally produced in the Ti^4+^ range between 10 and 100 mol% (see Figure 1). Based on the structural refinement analyses, we argue that the stability of the cubic structure is promoted by low-temperature crystallisation (125–200 °C); under these conditions, the octahedral BO_6_ units are aligned at centrosymmetric positions inside the cubic structure. Furthermore, the systematic variation of Ti-O and Zr-O bond lengths and the lattice strain (Table S3 in the Supplementary supporting information file) contribute to compensating for the global physical changes caused by the composition rather than provoking distortion of the BO_6_ units [9,11].

**Figure 5:**
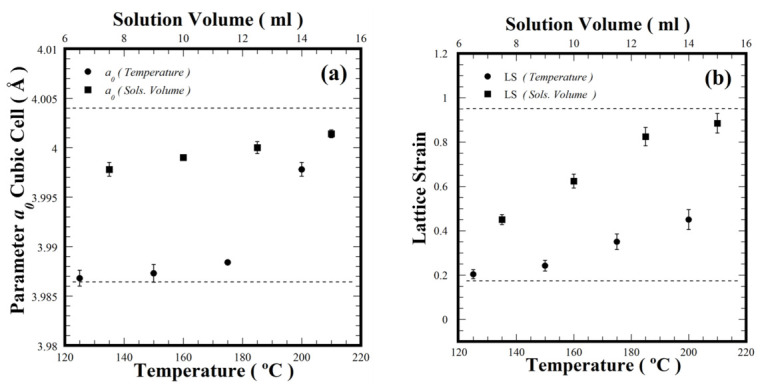
Crystalline lattice constant variation (**a**) “*a*_0_” and (**b**) residual lattice strain of perovskite structured SrTi_0.5_Zr_0.5_O_3_, prepared at standard conditions at 200 °C for 6 h and vigorous stirring at 130 rpm.

A detailed analysis of the experimental conditions (reaction temperature and metal precursor solution volume) was conducted to determine their effects on the dissolution of the coprecipitated gel and on the residual lattice strain in the structure. The SrTi_0.5_Zr_0.5_O_3_ SS1 was selected as the standard composition, and the variation of the residual lattice strain is shown in Figure 5. Generally, the reaction temperature affected the lattice constant “*a*_0_” of cubic SrTi_0.5_Zr_0.5_O_3_ above 175 °C; a remarkable increase of ∆a/a=0.02% was only observed in the sample synthesised at 200 °C, and no marked differences in “*a*_0_” at lower temperatures were determined. Interestingly, “*a*_0_” values of the powders produced with different nominal concentrations of the metal precursor at 200 °C did not exhibit remarkable differences, as seen in Figure 4a. Nevertheless, the change in the solvent bulk composition caused by varying the volume of metal solutions alters the dielectric constant and solvent heat capacity, thereby affecting the rate at which the dissolution-crystallisation mechanism proceeds. This phenomenon might induce differences in unit-cell lattice constants even in a single-phase compound produced under hydrothermal conditions [25]. The level of residual lattice strain in the cubic-structured SS1 was affected by the hydrothermal processing conditions, as seen in Figure 4b. According to structural refinement analyses (Table 1), the residual lattice strain increased by a factor of two, varying both the reaction temperature and the nominal metal solution volume. The lattice strain is likely induced by differences in the dissolution rate of the coprecipitated gel, which consequently accelerates the particle crystallisation stage under hydrothermal conditions, as depicted in Figure 4b. The systematic variation in residual lattice strain might trigger the transformation of the cubic structure in the hydrothermally prepared SS1. However, the calculated global structural strain level maintains the structural stability of the cubic unit cell, namely in SrZr_1−x_Ti_x_O_3_ SSs1 (10.0 ≤ x ≤ 50.0 mol% Ti^4+^). Even the maximum strain (0.88) calculated for the sample obtained at 200 °C, with a volume of 15 mL for each metal solution, does not exceed the required strain to break atomic bonds, which is one of the main causes for achieving crystalline structural transformation in solid materials [25].

### 2.3. Morphological Evolution of Cubic Perovskite SrZr_1−x_Ti_x_O_3_ and SrTi_1−x_Zr_x_O_3_ SS Prepared via Gel Coprecipitation Assisted by Hydrothermal Processing

**Figure 6:**
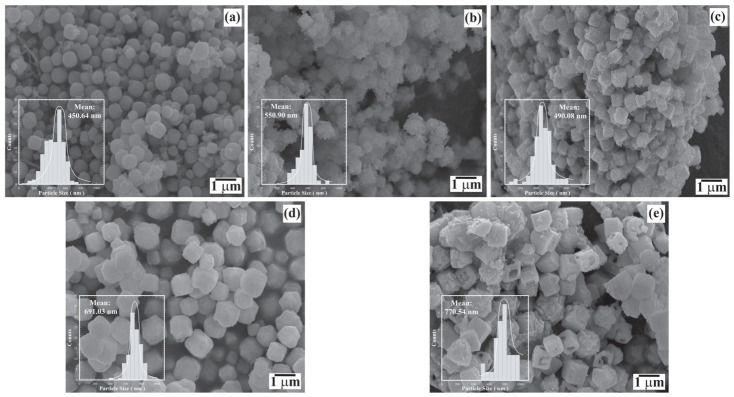
FE–SEM micrographs of reaction products of (**a**) SrTiO_3_, (**b**) SrTi_0.67_Zr_0.33_O_3_, (**c**) SrTi_0.473_Zr_0.527_O_3_, (**d**) SrZr_0.74_Ti_0.26_O_3_, and (**e**) SrZrO_3_, obtained with 5 M KOH at 200 °C for 6 h at 130 rpm.

Figure 6 shows the typical morphology of the cubic-structured particles synthesised via gel coprecipitation and hydrothermal crystallisation in the alkaline fluid with vigorous stirring. The micrographs in Figure 6 correspond to the single-phase SrTiO_3_ and SrZrO_3_, as well as the SS1 samples with intermediate compositions of SrTi_0.67_Zr_0.33_O_3_, SrZr_0.527_Ti_0.473_O_3_ and SrZr_0.74_Ti_0.26_O_3_. Interestingly, FE-SEM observation revealed that pseudo-cuboidal SrTiO_3_ particles with a high degree of edge truncation were produced by hydrothermal crystallisation. These particles exhibited a highly monodisperse distribution, with an average particle size of 469.0 ± 6.8 nm (Table S3, Figure 6a). However, a slight change in particle morphology resulted in the cubic-perovskite (SS1) SrTi_1−x_Zr_x_O_3_ and SrZr_1−x_Ti_x_O_3_ particles. The morphology of these particles resembled a euhedral cuboidal shape with right edges; this particle shape is irrespective of the amount of Ti^4+^ incorporated into the SS1s with a cubic structure. In contrast, the size of cuboidal particles slightly increased when the Zr^4+^ content varied from 52.7 to 100.0 mol%; the increment to an average value of 515.2 ± 6.1 nm (Figure 6b,c) occurred in the sample of SrZr_0.527_Ti_0.473_O_3_, which corresponds to the crystallite size determined by Rietveld refinement. A marked increase of 1.5-fold order took place in the particle size of the cuboidal-shaped Zr^4+^-rich (SrZr_1−x_Ti_x_O_3_) sample, as depicted in Tables S3 and S4; in fact, the maximum average particle size of 816.4 ± 4.3 nm was obtained on the sample of SrZrO_3_, as seen in Figure 6e. These values were used as a reference to evaluate particle microstructural differences, because these are nearly analogous to those calculated from linear measurements in FE-SEM micrographs for each sample, as summarised in Table S4.

**Figure 7:**
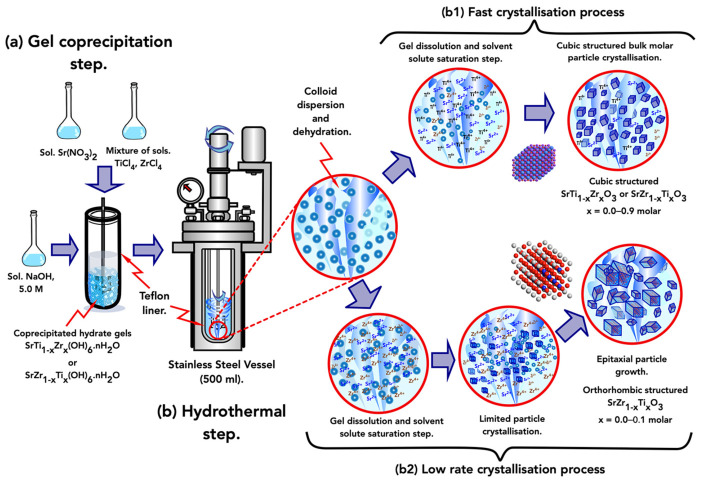
Schematic representation of the two-stage synthesis route used to prepare SrTi_1−x_Zr_x_O_3_ and SrZr_1−x_Ti_x_O_3_; (**a**) First step, precursor gel coprecipitation and subsequently (**b**) Hydrothermal crystallisation step of (**b1**) cubic fine perovskite particles and (**b2**) low rate crystallisation of orthorhombic large particles SrZrO_3_, obtained with 5 M KOH at 200 °C for 6 h at 130 rpm.

Figure 7 shows a schematic representation of the global process that drives particle synthesis and morphological differences among the selected perovskite compositions in the binary SrTiO_3_–SrZrO_3_ system. The first step involves the formation of a gel that is described by the chemical equilibria given in Equations (S1), (S4), (S7) and (S10) in the Supplementary supporting information file). This reaction rapidly occurs when a strong alkaline solution is added, resulting in the condensation of the inorganic metal hydroxide. The dispersion of the gel, provoked by continuous stirring, formed fine colloid aggregates homogeneously dispersed in the solvent alkaline media. Consequently, the gel’s aggregates rapidly dehydrate at the early stages of the hydrothermal treatment, irrespective of their chemical composition. We argue that since all the experiments were carried out at a constant stirring speed (130 rpm), the dispersed aggregate sizes did not vary markedly. Therefore, this process does not affect the main dissolution-crystallisation mechanism that controls the process’s second step. Subsequently, continuous dissolution of dehydrated aggregates takes place according to Equations (S3), (S6), (S9) and (S12) (Section S2), causing the solvent to supersaturate with metal ions at the selected stoichiometric ratio. This inference is supported because any secondary oxide phase related to the metal ions was produced during the hydrothermal treatment. At this stage, spontaneous embryo nucleation occurs when the alkaline liquor reaches a maximum supersaturation level [20,26]. In ordinary hydrothermal treatments, the larger the solute molar volume, the greater the supersaturation level achieved, leading to the nucleation of a vast number of embryos per unit molar volume in the fluid. Under this specific reaction pathway, particle growth is limited by the rapid precursor gel consumption, resulting in the crystallisation of finer particles (Figure 7(b1)). This reaction trend is likely to lead to the preparation of the SS1 perovskites in the 50.0–100.0 mol% Ti^4+^ range, driving the formation of particles with fine sizes ranging from 550.0 to 450 nm (Figure 6a–c). On the contrary, the reduction in the gel’s dissolution rate decreases the solvent supersaturation level, resulting in the nucleation of a reduced number of embryos. Simultaneously, the dissolution of the remaining gel aggregates continues to provide the solute, which progressively stacks at the embryo surfaces; see Figure 7(b2). Under this global hydrothermal steady state, the epitaxial growth mechanism proceeds, promoting a gradual increase in particle size rather than the nucleation of new embryos [20,26]. This assumption is consistent with the increase in particle size of SS1 Zr^4+^-rich SrZr_1−x_Ti_x_O_3_ with a cubic structure (10.0–50.0 mol% Ti^4+^, 691 nm) and orthorhombic-structured SrZrO_3_ (average particle size 770 nm) powders. Hence, our results suggest that the gel dissolution rate is affected by the zirconium content, owing to the higher chemical stability of the coprecipitated gel than that of the SS1 perovskites rich in titanium (SrZr_1−x_Ti_x_O_3_), provoking only a systematic size variation of the pseudocubical-shaped particles, as seen in Figure 6.

**Figure 8:**
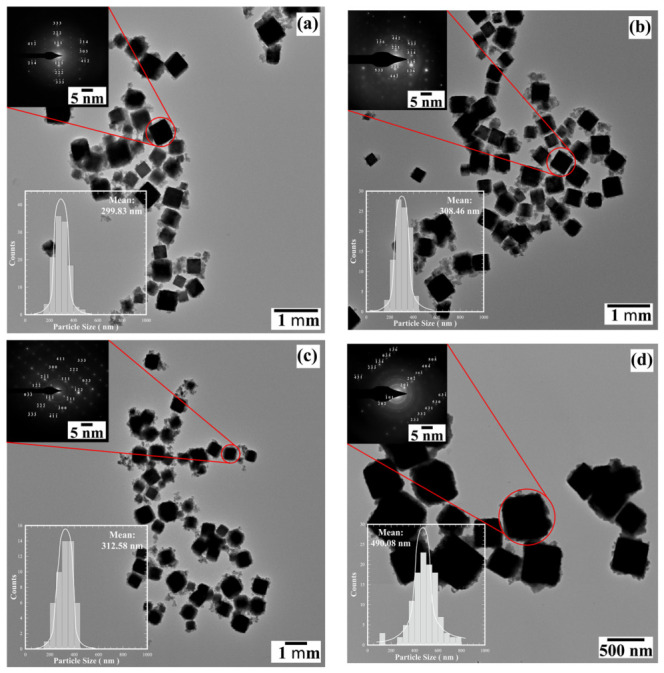
TEM micrographs of SrTi_0.5_Zr_0.5_O_3_ powders obtained under hydrothermal conditions with 5 M KOH solution for 6 h at temperatures of (**a**) 125 °C, (**b**) 150 °C, (**c**) 175 °C and (**d**) 200 °C.

In addition, detailed TEM analyses of the SrTi_0.5_Zr_0.5_O_3_ cuboidal particles showed that within the temperature range of 125–175 °C, the particle size varied between 299.83 ± 13.5 nm and 312.58 ± 9.8 nm even under vigorous stirring at 130 rpm, as revealed in Figure 8a–c. On the contrary, when the temperature increased to 200 °C, the SrZr_0.527_Ti_0.473_O_3_ perovskite SS1 had a remarkable increase in size, averaging 490.1 ± 10.3 nm. At this temperature, the dissolution-precipitation mechanism is slowed, and agitation causes rapid colloid break-up by fluid convection, contributing to epitaxial growth that coarsens the particles [26]. Additional structural observations of the cubic perovskite SrTi_0.5_Zr_0.5_O_3_ obtained by SAED analyses conducted on selected areas are indicated by the red circle in each micrograph. In all cases, the SAED images revealed typical diffraction spot patterns, demonstrating that the SrTi_0.5_Zr_0.5_O_3_ particles structurally correspond to a single crystal with excellent crystallinity (Figure 8). This crystalline aspect was also revealed in the SS1 particles with different compositions as shown in Figure S3 in the Supplementary supporting information file. Furthermore, the calculated spacing between crystalline planes (indexed spots in the SAED images) coincides with cubic symmetry. It is worth emphasising that the spots of the crystallographic superlattice distortions, which are correlated with tetragonal or orthorhombic structures, were not detected in all SAED images. Hence, these results confirmed that the usage of a coprecipitated gel favours the crystallisation of cubic perovskite-structured solid solutions within the range of 10.0–100.0% Ti^4+^; this result is supported by the displacement observed in the PXRD analyses in Figure 2. On the contrary, at 200 °C, SS1 particles grow via an epitaxial mechanism, achieved by vigorous stirring during the crystallisation stage (Figure 8d). This increased the particle size up to 490 nm and reduced the number of tiny particles compared with those produced at temperatures below 175 °C (Figure 8a–c).

**Figure 9:**
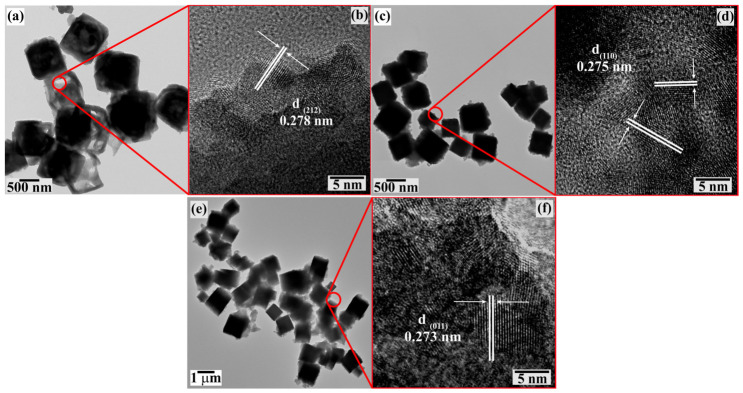
Images of TEM (**a**,**c**,**e**) and HR-TEM (**b**,**d**,**f**) micrographs of particles synthesised corresponding to (**a**,**b**) SrZrO_3_, (**c**,**d**) SrZr_0.527_Ti_0.473_O_3_ and (**e**,**f**) SrTiO_3_.

In addition, HR-TEM observations were conducted to investigate details of the pseudo-cuboidal particle surface, which exhibits distortions that affect the global particle morphology. The images of selected specimens corresponding to the single phases SrZrO_3_, SrTiO_3_, and the SrZr_0.527_Ti_0.473_O_3_ intermediate SS1 are shown in Figure 9. In general, the presence of fine irregular chunks preferentially occurred in the samples of SrZr_0.527_Ti_0.473_O_3_ (Figure 9c) and SrTiO_3_ (Figure 9e). Although these irregular aggregates might correspond to residual dehydrated gel (amorphous), the HR-TEM images confirmed that they are composed of tiny particles with an average particle size of 7.0 ± 2.0 nm, exhibiting high structural crystalline order (Figure 9b,d,f). These images suggest that the fine crystals spontaneously crystallised in an aleatory way rather than by the epitaxial growth mechanism argued to trigger the bulk particle coarsening. Furthermore, the 2D finger-lattice patterns indicate that the nanoparticles crystallise in a preferential crystallographic direction. Thus, the measured fringe spacing of 0.278 nm in Figure 9b demonstrates that the fine crystal forms preferentially along the {212} plane of the SrZrO_3_ orthorhombic structure, while the fringe spacings calculated as 0.273 nm and 0.275 nm for SrTiO_3_ and SrZr_0.527_Ti_0.473_O_3_ particles, respectively, indicate that the particle growth occurred along the {011} plane of the cubic structure. The small difference in the “d_(011)_” values is consistent with the slight peak displacement to low angles in the PXRD plotted in Figure 1, which is due to the unit cell expansion caused by the Zr^4+^ ion substituting Ti^4+^ in the cubic structure. These results confirm that the cubic structure is the unique phase that can be produced at low temperature (125–200 °C) by the processing approach investigated. Regarding the crystallisation of the fine, irregular agglomerates, we argue that the residual solute saturating the solvent rapidly crystallised during the cooling stage because it proceeded dynamically under vigorous stirring. Therefore, under these conditions, the solute supersaturation decreased, yielding a large embryo molar volume that exhibited minimal size increase. The agglomeration of the nanosized particles triggered the formation of fine agglomerates distributed randomly on the particle surfaces.

### 2.4. Optical and Band Gap Evaluation of Cubic Perovskite SrTi_1−x_Zr_x_O_3_ and SrZr_1−x_Ti_x_O_3_ SSs Prepared via Gel Coprecipitation Assisted by Hydrothermal Processing

**Figure 10:**
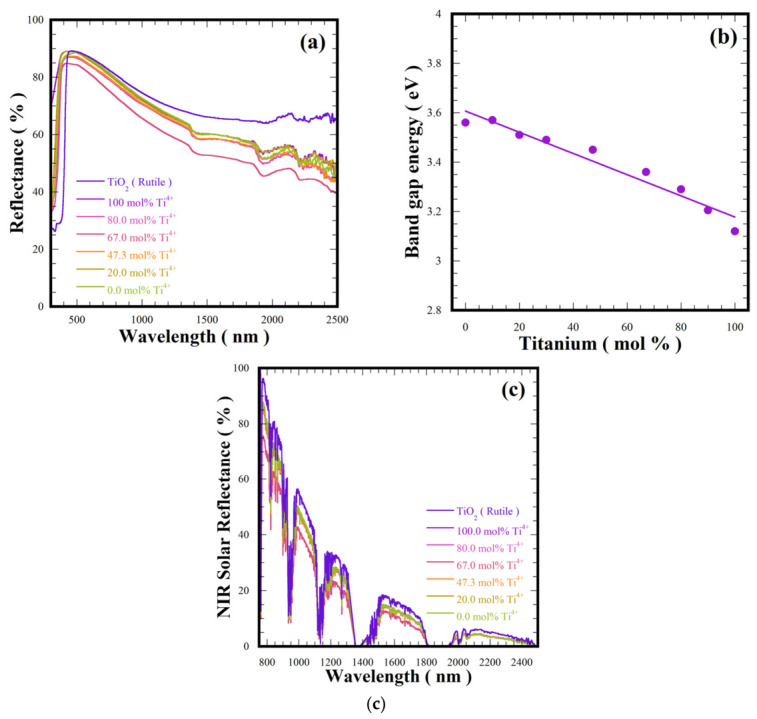
(**a**) Near infrared reflectance (NIR) spectra, (**b**) variation of the direct band gap energy and (**c**) solar reflectance spectra of SrTiO_3_, SrTi_1−x_Zr_x_O_3_, SrZr_1−x_Ti_x_O_3_ and SrZrO_3_ powders, respectively.

The optical properties of the single-phase SrTiO_3_, SrZrO_3_ and cubic SS1 powders prepared by the proposed coupled gel coprecipitation hydrothermal processing are portrayed in Figure 10. An emphasis on investigating CIELab colour parameters, diffuse reflectance, solar irradiance, and band gap energy was conducted for all the samples prepared with various Ti^4+^ contents by UV–Vis NIR spectrometry. Therefore, band gap energy measurements were carried out on the cubic SS1 compounds. This evaluation is among the most accurate within the proposed standard methodologies proposed for predicting the potential technological applications of a wide variety of oxide materials, even though no preliminary testing evaluation is provided. In addition, the TiO_2_ powder (Aldrich, 99.9% purity, particle size of 5.0 μm) was selected as a standard pigment for colour and diffuse reflectance measurements. Furthermore, the solar irradiance was calculated from the diffuse reflectance curves according to the ASTM G173-03 standard [27]. The details of the measurements carried out in the range of 750–2500 nm are included in Section S4 of the Supplementary supporting information file. Furthermore, the colour parameters (CIELab coordinates, chroma (Cab*), colour hue), solar irradiance (%R*) and band gap are summarised in Table S4 in the Supplementary supporting information file. Generally, the colour of SrTiO_3_ and SrZrO_3_ powders is similar to that of the SS1s, and the colour tonality falls within the light grey space, with chroma values ranging from 1.19 to 1.73. However, the TiO_2_ (1.67) chroma falls within this range and exhibits RGB colour coordinates (251, 249, 246) that approach the pure white colour hue (255, 255, 255) [28,29].

Figure 10 shows the typical diffuse reflectance curves of the TiO_2_ powders and those of the selected powders hydrothermally prepared using a coprecipitated gel. Interestingly, the single-phase SrTiO_3_, SrZrO_3_, and cubic SS1 powders exhibited a high spectral reflectance in the UV and Vis regions. This behaviour is depicted by a sharp increase up to a value of 89.0%, which remained constant up to a wavelength of 650 nm. On the contrary, the standard TiO_2_ reflectance is limited in the UV region, reaching approximately 89.0% at 400 nm, and remains constant up to 700 nm in the Vis region, as seen in Figure 10a. Although the TiO_2_ reflectance in the UV-Vis region has been reported to be high, the value is lower than that of the hydrothermally prepared SS1 and SS2 perovskite powders. We argue that this difference might be due to the powder particle size (as seen in Table S3), which is among the chief parameters affecting the pigment optical properties [28,29].

On the other hand, the diffuse reflectance in the UV-Vis region was used to experimentally assess the band gap of powdered cubic (SS1) and orthorhombic (SS2) structured hydrothermally synthesised powders. The direct band gap measurement was selected based on the clear linear region observed in the Tauc plots calculated using the Kubelka–Munk method, as shown in Figure S4 in the Supplementary supporting information file. To corroborate the accuracy of the analysis, Tauc’s indirect band gap was calculated; the resulting curves did not exhibit clear regions associated with the absorption band edge, and the lines did not start at zero on the y-axis, which is mandatory for indirect band gap selection [30]. This optical property was considered to estimate the potential applications of SS1 and SS2 perovskite powders [31]. Furthermore, the direct band gap values of the perovskite powders, “*E_g_*”, decreased markedly with increasing Ti^4+^ content in the intermediate cubic SS1s, as seen in Figure 10b. The correlation coefficient, R^2^, for these data was 0.96, indicating that the *E_g_* decrement is proportional to the Ti^4+^ content that substitutes for Zr^4+^ at the B site of the cubic ABO_3_ perovskite. According to the systematic decrease in the direct band gap from 3.57 eV (SrZrO_3_) to 3.12 eV (SrTiO_3_). We surmise that the narrowing in *E_g_* might be caused by structural distortions that alter Ti-O and Zr-O bond lengths, coupled with lattice strain, rather than by particle-size reduction. These structural distortions might be caused by localised changes in the electronic states of the Zr-4d, Ti-3d, and O 2p orbitals. Thus, the Zr-4p electronic hybridisation is likely to proceed on the SS1 and is attenuated by Ti-3d and O-2p orbitals induced by ion doping. This inference is supported by the congruent decrease in the bond length distances and the residual lattice strain calculated by crystalline refinement analysis (Table S3 in the Supplementary supporting information file) [32,33,34,35]. The perovskite-structured materials developed by gel coprecipitation assisted by hydrothermal processing exhibit a wide *E_g_* value range (3.12–3.6 eV), indicating they exhibit optimal absorption in the near-UV to UV region. Moreover, the typical direct band gaps of pure SrTiO_3_ and SrZrO_3_ (170.6 nm) submicron-sized powders are 3.2 eV and 5.65 eV, which were obtained by sol–gel calcination (700 °C) [36] and high-temperature solid-state reaction (1400 °C) [37], respectively. In comparison, the hydrothermally prepared fine powders, SrTiO_3_ (450.6 nm) and SrZrO_3_ (770.5 nm), have lower values: 3.12 eV and 3.57 eV, respectively. The marked difference in bandgap values, namely for SrZrO_3_, is not attributed to the powder particle size; on the contrary, the high-temperature processing (1400 °C) must induce a remarkable level of structural defects that hinder the band gap narrowing. However, a controversy arises regarding the perovskite solid solution; the simultaneous substitution of Sr (15 mol%) and Zr (15%) in BaTiO_3_, yielding Ba_0.85_Sr_0.15_Ti_0.85_Zr_0.15_O_3_ obtained at 1450 °C, reduced the direct band gap to 2.41 eV, lower than that of the cubic solid solutions synthesised at 200 °C. Based on our results, we surmise that the improvement in optical performance reflects the main novelty of the processing approach’s effectiveness. Additionally, the findings provide insights for defining the potential applications of the compounds prepared in the binary system SrTiO_3_–SrZrO_3_. Hence, these compounds can be employed to develop optoelectronic devices, such as selective photodetectors or gas-sensing detectors [31,33]. An additional application exists in the field of UV-active photocatalysis, particularly for water-splitting reactions that produce H_2_ or O_2_ [34,35].

Additional optical characterisation was carried out to evaluate the feasibility of potential applications as energy-saving and environmentally friendly cool pigment coatings, characterising the NIR solar irradiance (%R*) performance of the prepared compounds. The solar irradiance was calculated in the wavelength range of 750–2500 nm, and typical curves for selected compositions in the binary system SrTiO_3_–SrZrO_3_ are shown in Figure 9c; the values of %R* are summarised in Table S4. It is worth emphasising that the perovskite compounds exhibit remarkable solar irradiance behaviour, which is analogous to that of the TiO_2_ standard in the NIR region. The values of this optical property fluctuate within the range between 77.6% and 86.49%, and particle size measurements indicate that the %R* depends on particle size rather than morphology (see Table S4). The solar irradiance capability of the perovskite produced by the chemical process investigated is analogous to that of several oxides designated as cool pigments with different colours, specifically, white coloured titania (91.0%) and LiAlW_2_O_8_ (92.13%) [38], yellow SrBaCe_0.6_Tb_0.4_O_4_ (91.0%) [39] and YIn_0.9_Mn_0.1_O_3_ (90.0%) [40], and red Ba_2_Ce_0.6_Tb_0.4_O_4_ (89.0%) [39] and perovskite YAlO_3_ co-doped with Cr and Mg (73.4–89.9%) [40], among others [41]. Hence, the perovskite orthorhombic and cubic structured (SS1) solid solutions in the SrTiO_3_–SrZrO_3_ system have the potential to develop cool coatings for energy-saving and environmentally friendly applications in the building construction field. The use of cool coatings might reduce air-conditioning and cooling power consumption due to their high solar reflectance.

## 3. Conclusions

The systematic investigation aimed to broaden the chemical stability of cubic structured perovskite SSs in the binary SrTiO_3_–SrZrO_3_ system was successfully carried out through chemical processing comprising in situ gel coprecipitation and hydrothermal crystallisation. The coprecipitated gel dehydration step proceeded rapidly; thus, the dissolution of the dehydrated gel occurred at a constant steady state, triggering an accelerated release of the cationic species according to the selected nominal molar ratio. Hence, the supersaturation of the alkaline media resulted in the crystallisation of cubic-structured SrTi_1−x_Zr_x_O_3_ and SrZr_1−x_Ti_x_O_3_ solid solutions, extending nearly to the entire intermediate compositional range of 10.0–100 mol% Ti^4+^. The crystallisation was boosted by vigorous stirring of the 5 M KOH solution and occurred at 125 °C for 6 h. It is noteworthy to emphasise that all the powders prepared exhibited a pseudocuboidal-shaped morphology, even the orthorhombic SrZrO_3_ powders. This morphology agrees with the structural habit of the cubic perovskite SrTiO_3_. The gradual increase in Ti^4+^ content leads to a marked reduction in particle size, from 980.0 nm (SrZrO_3_) to 450.6 nm (SrTiO_3_). Therefore, at high Ti^4+^ contents (<50.0 mol%), the rate of dissolution of the gel slowed down, decreasing the embryo molar volume in the solvent medium (KOH), causing the epitaxial growth, which promotes the particle coarsening in Zr^4+^-rich SrZr_1−x_Ti_x_O_3_ SSs.

Additionally, the optical performance analysis of the perovskite powders in the UV–Vis–NIR spectrum demonstrates their potential functional applications. Although these powders exhibit a light grey hue (L* = 93.0), their colour is close to the TiO_2_ standard white pigment (L* = 93.0). Likewise, the direct band gap (*E_g_*) of these perovskite compounds varies in a narrow range between 3.12 eV and 3.57 eV. This property can be tuned by the Ti^4+^ content incorporated in the structural B site rather than the particle size reduction. These findings provide relevant experimental insights into the functional applications of perovskite materials as semiconductors and high-UV photocatalysts. Furthermore, the high solar irradiance (%R*), averaging 80.22%, determined from the NIR spectrum (750–2500 nm) indicates that it can be used as a cool pigment for roof coating development. We believe that the novel sol–gel coprecipitation assisted by the investigated hydrothermal processing can be employed for other binary perovskite oxide systems to tailor solid solutions with enhanced functional properties.

## 4. Materials and Methods

### 4.1. Materials and Preparation of Precursor Coprecipitated Gels

Zirconium IV (ZrCl_4_) and titanium IV (TiCl_4_) reagent-grade chlorides and Sr(NO_3_)_2_•6H_2_O (Fujifilm Wako Pure Chemical Corporation, Osaka, Japan, 99.0% purity) were selected to produce the coprecipitated gels. Initially, the transition-metal (Zr^4+^ and Ti^4+^) and Sr^2+^ mother solutions were prepared at a concentration of 0.43 M in deionised water [20]. The KOH reagent-grade chemical (Fujifilm Wako Pure Chemical Corporation, Osaka, Japan), 99.0% purity) was used to prepare a 5 M solution. Experiments were carried out using this solution at different volumes between 25.0 and 55.0 mL.

### 4.2. Hydrothermal Treatments

Experiments focused on determining the feasibility of preparing stable single-phase SrZr_1−x_Ti_x_O_3_ and SrTi_1−x_Zr_x_O_3_ via a coupled gel coprecipitation and hydrothermal processing. The proposed chemical processing is likely to trigger a dissolution steady state of the gel coprecipitated during the hydrothermal treatment, resulting in the rapid crystallisation. The analysis aims to elucidate the crystalline structure and morphological features of the solid solutions (SSs), which may vary with the dissolution rate of the coprecipitated gel. Initial experiments were conducted to produce SSs across the entire compositional range of the binary SrTiO_3_–SrZrO_3_ system. Moreover, particular emphasis was placed on the range of 10.0–100.0 mol% of Ti^4+^ by systematically varying the molar span every 10 mol% of Ti^4+^, because the perovskite tetragonal structure has been reported to be thermodynamically stable at temperatures lower than 400 °C in this range [12,21]. Furthermore, the SSs with a content of 40.0 mol% (Zr^4+^ or Ti^4+^) were not investigated because further analyses were conducted on the intermediate perovskite SS1 SrZr_0.5_Ti_0.5_O_3_, which incorporated the maximum content of both metal cations at the perovskite structure B site. Hence, the second experimental run aimed to investigate the effect of experimental parameters, such as the reaction temperature (125–200 °C) and the volume of the metal liquor solutions (7.5–15.0 mL). All hydrothermal treatments were conducted in a vertical stainless steel batch reactor with an inner volume of 500 mL. This device is equipped with a stirring unit. Equimolar volumes of each metal ionic solution were calculated following the Sr:1−xZr:xTi molar ratio of 1:1−x:x. In a typical run, the solution volume for each metal solution selected was 15 mL, and the total volume of mother mixed liquor added to the Teflon vessel was 30 mL. Subsequently, the KOH solution with a concentration of 5 M (55 mL) was added to coprecipitate the milky white colloid (gel). The autoclave was then sealed and heated at a constant rate of 5 °C/min up to the desired temperature. The treatment was conducted with a reaction interval of 6 h, and the vertical propeller rotation speed was set to 130 rpm during the heating and soaking stages. At this rotation speed, a stable vortex hinders colloid ejection from the solution, as suggested elsewhere [20]. After treatment, the reaction products were separated from the remaining mother liquor and ultrasonically washed several times with hot distilled water to prevent K^+^ ions from adsorbing onto the particle surface. The powders were then dried at 80 °C overnight.

### 4.3. Characterisation

Powder X-ray diffraction (PXRD). The analyses were conducted on a Rigaku Ultima IV diffractometer (Rigaku Corporation, Tokyo, Japan) operated at 40 kV and 20 mA with Cu Kα radiation (*λ* = 1.54056 Å). Each sample was scanned over the 2θ range of 5–80° at a constant speed of 2°/min with a 0.02° step size. Furthermore, the crystalline structural analyses were carried out using Rietveld refinement on the PXRD patterns recorded in the 2θ range of 15–130°, with a scanning speed of 0.01°/min and a step size of 0.002°. The refinement calculation was performed using TOPAS 4.2 (v2013, Bruker AXS, Karlsruhe, Germany, 2009). The space group and the atomic spatial positions (Wyckoff numbers and coordinates) were taken from the ICDD cards Nos. 40-1500 and 70-0283, corresponding to the cubic (*Pm3m*) and orthorhombic (*Pbnm*) perovskites (see Tables S1 and S2 in the Supplementary supporting information file), respectively. All details associated with the refinement algorithm parameter are described in the supplementary supporting information file (SSIF). Section S1 includes Tables S1 and S2, where the Wyckoff spatial coordinates are given.

Morphology and microstructural observation. The microstructural features of the particles were observed using field-emission scanning electron microscopy (JEOL Ltd., Tokyo, Japan, JSM-7600F) and a solid-state microprobe (EBSD, JEOL Ltd., Tokyo, Japan). Typical micrographs were recorded at 10 kV, with a constant filament current of 69 μA. The particle size distribution was statistically determined from SEM images of 75 particles. Crystalline structural details of selected SrZr_1−x_Ti_x_O_3_ particles were revealed using high-resolution transmission electron microscopy (HR-TEM, SCIOS Talos F200X, Thermo Fisher Scientific Inc., Waltham, Massachusetts, USA) at 200 kV.

Optical properties and band gap measurements. The CIELab* coordinates and reflectance spectra of powders were measured using a UV–Visible–NIR spectrometer (Jasco V-770, Hachioji, Tokyo, Japan) equipped with an integrating sphere device. The baseline was measured using the BaSO_4_ plate as a calibration reference, and the spectrometer colour space parameters were measured according to the standard CIELab* colourimetry method. Additional details on the powder’s band-gap measurement, the chromatic parameters and the solar reflectance of the pigments are described in detail in Section S4 in the Supplementary supporting information file.

## Figures and Tables

**Table 1:** Summary of the hydrothermal treatment conditions selected to investigate the formation of SrZr_1−x_Ti_x_O_3_ and SrTi_1−x_Zr_x_O_3_ SSs from coprecipitated gel precursors.

Sample ID	TiCl_4_ (mL)	ZrCl_4_ (mL)	Sr(NO_3_)_2_ (mL)	KOH (mL)	Temperature (°C)	Chemical Formula	Crystalline Structure	Lattice Parameters			Lattice Strain	*R_wp_* (%)	*R_p_* (%)	*R_exp_* (%)	*R_Bragg_* (%)	GOF (χ^2^)
								a_0_ (Å)	b_0_ (Å)	c_0_ (Å)						
SZGG7	15	0	15	55	200	SrTiO_3_	Cubic	3.9133 (6)	-	-	−0.15 (3)	6.42	5.9	1.50	1.55	4.1
SZGG6	13.5	1.5	15	55	200	SrTi_0.9_Zr_0.1_O_3_	Cubic	3.9275 (4)			−0.07 (6)	6.16	4.79	1.42	1.35	4.3
SZGG16	12	3	15	55	200	SrTi_0.8_Zr_0.2_O_3_	Cubic	3.9380 (5)			0.18 (9)	4.48	5.13	1.31	1.61	3.6
SZGG5	10.5	4.5	15	55	200	SrTi_0.67_Zr_0.33_O_3_	Cubic	3.9585 (4)			−0.09 (6)	4.62	3.61	1.22	1.44	3.7
SZGG1	7.5	7.5	15	55	200	SrZr_0.527_Ti_0.473_O_3_	Cubic	3.9979 (7)			0.44 (5)	7.93	5.83	1.36	0.844	5.8
SZGG4	4.5	10.5	15	55	200	SrZr_0.74_Ti_0.26_O_3_	Cubic	4.0802 (15)			0.80 (8)	4.48	3.41	1.16	1.005	3.8
SZGG3	3	12	15	55	200	SrZr_0.8_Ti_0.2_O_3_	Cubic	4.0839 (8)			−0.42 (6)	3.98	3.09	1.12	0.547	3.8
SZGG2R	1.5	13.5	15	55	200	SrZr_0.9_Ti_0.1_O_3_	Cubic	4.0903 (10)			−0.44 (5)	3.86	3.03	1.07	0.468	3.4
SZGG8	0	15	150	55	200	SrZrO_3_	Orthorhombic	5.8082 (13)	5.8037 (38)	8.2129 (12)	0.46 (3)	4.49	3.32	1.04	1.56	4.5
SZGG12	15	15	30	25	200	SrZr_0.5_Ti_0.5_O_3_	Cubic	4.0034 (4)	-	-	0.88 (3)	5.27	4.34	1.17	1.37	5.0
SZGG11	12.5	12.5	25	35	200	SrZr_0.5_Ti_0.5_O_3_	Cubic	4.0010 (6)			0.82 (1)	6.14	4.48	1.20	2.55	4.6
SZGG10	10	10	20	45	200	SrZr_0.5_Ti_0.5_O_3_	Cubic	3.9996 (3)			0.62 (2)	12.72	9.50	1.44	1.177	8.8
SZGG13	7.5	7.5	15	55	175	SrZr_0.5_Ti_0.5_O_3_	Cubic	3.9887 (2)			0.35 (1)	4.02	3.11	1.12	1.853	3.3
Strontium titanate (tousinite) ICCD card No. 40-1500						SrTiO_3_	Cubic	3.905			-	-	-	-	-	-
Strontium zirconate ICDD card No. 70-0283						SrZrO_3_	Orthorhombic	5.786	5.815	8.19	-	-	-	-	-	-

## Data Availability

Data are contained in the article and are available upon request.

## Supplementary Materials

The following supporting information can be downloaded at: https://www.mdpi.com/article/10.3390/gels12060505/s1, Sections: S1. Rietveld Refinement details, refinement plots and selected orthorhombic and cubic unit cell lattices, S2. Chemical equilibria promoted in alkaline hydrothermal conditions for synthesising the cubic structured solid solutions in the binary system SrZrO_3_–SrTiO_3_, S3. Microstructural features of the SS particles prepared under alkaline hydrothermal conditions with vigorous fluid stirring, S4. Optical characterisation of cubic and orthorhombic SS particles prepared by coprecipitation and hydrothermal processing, Table S1: The atomic coordinates of SrTiO_3_ with a cubic structure (space group *Pm*$\bar{3}$*m*, 221) were used to carry out the Rietveld refinements with TOPAS 4.2 software; the spatial locations were reported previously in a CIF file, ICDD card No. 40-1500, Table S2: Strontium zirconate orthorhombic structure atomic coordinates (space group *Pbnm*, 62) were used to carry out Rietveld refinements with TOPAS 4.2 software; spatial locations were reported in a CIF file, ICDD card No. 70-0283, Figure S1: Selected Rietveld refinement plots of powder samples prepared at 200 °C for 6 h with 5 M KOH stirred at 130 rpm, using different Ti^4+^-gel contents of (a) 100, (b) 90.0, (c) 80.0, (d) 50.0, (e) 20.0, (f) 10.0 mol% Ti, respectively, Table S3: Summary of experiments conducted to investigate the stability of the cubic perovskite structure in SrTi_1−x_Zr_x_O_3_ and SrZr_1−x_Ti_x_O_3_ SSs under hydrothermal conditions for 6 h in 5 M KOH solution with vigorous agitation at 130 r.p.m., Figure S2: FE-SEM micrographs of particles of the solid solutions SrTi_1−x_Zr_x_O_3_ and SrZr_1−x_Ti_x_O_3_, obtained under hydrothermal conditions at 200 °C for 6 h, using a 5 M KOH and cationic solutions with an equimolar volume of 7.5 mL, with different contents of Ti^4+^ (a) 90.0, (b) 80.0, (c) 70.0, (d) 30.0, (e) 20.0, (f) 10.0 mol%, Figure S3: TEM micrographs of powders corresponding to the SSs SrTi_1−x_Zr_x_O_3_ and SrZr_1−x_Ti_x_O_3_, obtained under hydrothermal conditions at 200 °C for 6 h, using a 5 M KOH and cationic solutions with equimolar volume of 7.5 mL, with different contents of Ti^4+^ (a) 0.0, (b) 30.0, (c) 70.0, (d) 100.0 mol%, Table S4: Summary of the particle sizes, bandgap energy, solar reflectance, CIEL*a*b* colour parameters, chroma, RGB values, and observed colour hue SrZr_1−x_Ti_x_O_3_ and SrTi_1−x_Zr_x_O_3_ powders synthesised under hydrothermal conditions with 5 M KOH solution, Figure S4: Kubelka–Munk curves of perovskite powders prepared with various contents of Ti^4+^ under hydrothermal conditions at 200 °C for 6 h with a 5 M KOH solution stirred at 130 r.p.m.

## Abbreviations

The following abbreviations are used in this manuscript:

PXRD	Powder X-ray diffraction
SS1	Cubic structured solid solutions of SrTi_1−x_Zr_x_O_3_ or SrZr_1−x_Ti_x_O_3_
SS2	SrZr_1−x_Ti_x_O_3_ orthorhombic solid solution
GOF	Goodness-of-fit factor
FE-SEM	Field emission scanning electron microscopy
SAED	Selected area electron diffraction
TEM	Transmission electron microscopy
HR-TEM	High resolution-transmission electron microscopy
NIR	Near infrared spectrum
CIELab	International Commission on Illumination
ASTM	American Society for Testing and Materials
