# Supplementary material for: Coupled Gel Coprecipitation and Hydrothermal Processing to Synthesise Cubic Structured Compounds in the SrTiO3–SrZrO3 System
Source: Gels. 2026 Jun 6;12(6):505. doi: 10.3390/gels12060505 (PMC13298226; doi:10.3390/gels12060505)
Supplement: Supplementary file 1 [file gels-12-00505-s001.zip › gels-4265597-supplementary.pdf]

Supplementary Supporting Information

Coupled gel coprecipitation and hydrothermal processing to synthesise cubic structured compounds in the $SrTiO_3$–$SrZrO_3$ system

Juan Carlos Rendón-Angeles[1,], Zully Matamoros-Veloza[2], Diego Emiliano Carrillo-Ramírez[1], José Remigio Quiñones-Gurrola[1], Kazumichi Yanagisawa[3]*

[1] *Centre for Research and Advanced Studies of the National Polytechnic Institute, Saltillo Campus, Ramos Arizpe, 25900, Coahuila, México; jcarlos.rendon@cinvestav.edu.mx, diego.carrillo@cinvestav.mx*

[2] *Tecnológico Nacional de México (I.T. Saltillo), Technological Institute of Saltillo, Graduate Division, Saltillo 25280, México; zully.mv2@saltillo.tnm.mx.*

[3] *Research Laboratory of Hydrothermal Chemistry, Faculty of Science, Kochi University, Kochi 780-8073; Japan; yanagi@kochi-u.ac.jp*

* *Correspondence: jcarlos.rendon@cinvestav.edu.mx (J.C.R.A.); Tel.: +52 (844) 438 9600*

This document contains supplementary data on the Rietveld refinement. Likewise, morphological observations of the solid solutions $SrTi_{1-x}Zr_xO_3$ and $SrZr_{1-x}Ti_xO_3$ conducted using SEM are presented in this document to support the analysis of morphology and crystallisation.

S1. Rietveld Refinement details, refinement plots and selected orthorhombic and cubic unit cell lattices.

The crystalline spatial coordinates and Wyckoff data for the phases indexed from the XRD patterns of the reaction products are shown in Tables S1 and S2. These cards are from the COD 2014 database, which runs with the High Score Plus Panalityca software 3.0e. These data were included in a subroutine of the algorithm program used for structural refinement analysis. The XRD pattern background in the algorithm was modelled using a 10-coefficient shifted Chebyshev polynomial, and the pseudo-Voigt function was selected to match the peak shape profile. The algorithm calculates the unit lattice cell parameters, the isotropic thermal displacement, the crystallite size, and M-O bond lengths (M = Sr^{2+}, Ti^{4+}, Zr^{4+}). Typical plots obtained by the refinement algorithm that considers the reaction by-product phases are portrayed in Fig. S1.

Table S1. The atomic coordinates of $SrTiO_3$ with a cubic structure (space group $Pm\bar{3}m$, 221) were used to carry out the Rietveld refinements with TOPAS 4.2 software; the spatial locations were reported previously in a CIF file, ICDD card no. 40-1500.

Element identification	Wyckoff position	Occupation	Spatial coordinates ($SrTiO_3$)		
			x	y	z
Sr	4c	1	0.5	0.5	0.5
Zr	4a	1-x	0.0	0.0	0.0
Ti	4a	x	0.0	0.0	0.0
O	8c	1	0.5	0.0	0.0

Table S2. Strontium zirconate orthorhombic structure atomic coordinates (space group *Pbnm*, 62) were used to carry out Rietveld refinements with TOPAS 4.2 software; spatial locations were reported in CIF file ICDD card no. 70-0283.

Element identification	Wyckoff position	Occupation	Spatial coordinates ($SrZrO_3$)		
			x	y	z
Sr	8d	1	0.9916	0.0123	0.25
Zr	4c	1	0.5	0.0	0.0
O1	4b	1	0.0586	0.4687	0.25
O2	4a	1	0.713	0.288	0.0371

Plots calculated from the structural Rietveld refinement analyses:

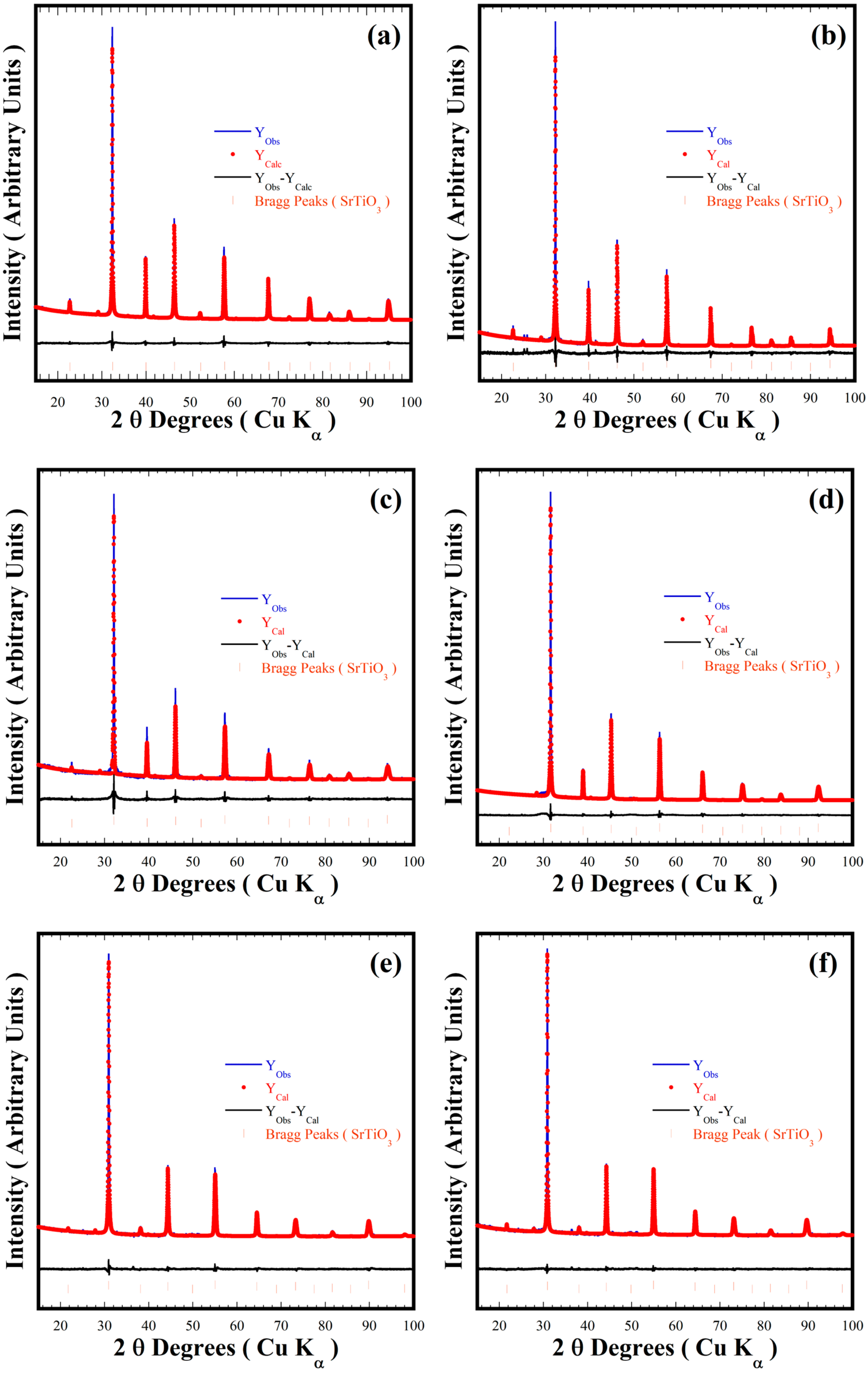

Figure S1. Selected Rietveld refinement plots of powder samples prepared at 200 ºC for 6 h with 5 M KOH stirred at 130 rpm, using different Ti^{4+}-gel contents of (a) 100, (b) 90.0, (c) 80.0, (d) 50.0, (e) 20.0 (f) 10.0 mol% Ti, respectively.

S2. Chemical equilibria promoted in alkaline hydrothermal conditions for synthesising the cubic structured solid solutions in the binary system $SrZrO_3$–$SrTiO_3$.

The chemical equilibria associated with the new synthesis process investigated were proposed from PXRD analyses of the reaction products prepared within the entire compositional range of the binary $SrTiO_3$–$SrZrO_3$ system; the equilibria are depicted by Eqs. S1-S4. In all the equilibria derived for the one-step reaction, the first equation corresponds to the preparation of the sol-gel precursor (Eq. Xa), while the second reaction involves gel dehydration (Eq. Xb), prior to the final step, where the dehydrated gel dissolution-crystallisation occurs, which triggers the formation of nanosized particles under alkaline hydrothermal conditions. This process occurred in a single step promoted by the continuous hydrothermal media stirring at 130 rpm. The sub-index abbreviations in the chemical equilibria, "ort" and "cub", correspond to orthorhombic and cubic perovskite crystalline structures.

$SrNO_{3(aq)} + TiCl_{4(aq)} + 5KOH_{(aq)}$

$\rightarrow SrTi(OH)_6{\bullet}nH_2O_{(gel)} + KOH_{(aq)} + KNO_{3(aq)} + 4KCl_{(aq)} + yH_2O$ (S1)

$\rightarrow SrTi(OH)_{6(gel)} + KOH_{(aq)} + KNO_{3(aq)} + 4KCl_{(aq)} + mH_2O$, where m = n+y (S2)

$\rightarrow SrTiO_{3(s,cubic)} + KOH_{(aq)} + KNO_{3(aq)} + 4KCl_{(aq)} + mH_2O$ (S3)

Where x = 50.0–100.0 mol% Ti^{4+}

$SrNO_{3(aq)} + (1\text{-}x)TiCl_{4(aq)} + xZrCl_{4(aq)} + 5KOH_{(aq)}$

$\rightarrow SrTi_{1\text{-}x}Zr_x(OH)_6{\bullet}nH_2O_{(gel)} + KOH_{(aq)} + KNO_{3(aq)} + 4KCl_{(aq)} + yH_2O$ (S4)

$\rightarrow SrTi_{1\text{-}x}Zr_x(OH)_{6(gel)} + KOH_{(aq)} + KNO_{3(aq)} + 4KCl_{(aq)} + yH_2O$, where m = n+y (S5)

$\rightarrow SrTi_{1\text{-}x}Zr_xO_{3(ss,cubic)} + KOH_{(aq)} + KNO_{3(aq)} + 4KCl_{(aq)} + mH_2O$ (S6)

Where x = 10.0 – 50.0 mol% Ti^{4+}

$SrNO_{3(aq)} + (1\text{-}x)ZrCl_{4(aq)} + xTiCl_{4(aq)} + 5KOH_{(aq)}$

$\rightarrow SrZr_{1\text{-}x}Ti_x(OH)_6{\bullet}nH_2O_{(gel)} + KOH_{(aq)} + KNO_{3(aq)} + 4KCl_{(aq)} + yH_2O$ (S7)

$\rightarrow SrZr_{1\text{-}x}Ti_x(OH)_{6(gel)} + KOH_{(aq)} + KNO_{3(aq)} + 4KCl_{(aq)} + mH_2O$, where m = n+y (S8)

$\rightarrow SrZr_{1\text{-}x}Ti_xO_{3(ss,cubic)} + KOH_{(aq)} + KNO_{3(aq)} + 4KCl_{(aq)} + mH_2O$ (S9)

$SrNO_{3(aq)} + ZrCl_{4(aq)} + 5KOH_{(aq)}$

$\rightarrow SrZr(OH)_6{\bullet}nH_2O_{(gel)} + KOH_{(aq)} + KNO_{3(aq)} + 4KCl_{(aq)} + yH_2O$ (S10)

$\rightarrow SrZr(OH)_{6(gel)} + KOH_{(aq)} + KNO_{3(aq)} + 4KCl_{(aq)} + mH_2O$, where m = n+y (S11)

$\rightarrow SrZrO_{3(s,ort)} + KOH_{(aq)} + KNO_{3(aq)} + 4KCl_{(aq)} + mH_2O$ (S12)

Table S3. Summary of experiments conducted to investigate the stability of the cubic perovskite structure in $SrTi_{1-x}Zr_xO_3$ and $SrZr_{1-x}Ti_xO_3$ SSs under hydrothermal conditions for 6 h in 5 M KOH solution with vigorous agitation at 130 r.p.m.

Sample ID	Ti % at	Zr % at	Sr % at	Temperature (°C)	Solution Volume Ti (ml)	Crystalline Phases	Crystalline Structure	Lattice Parameters[a]			Lattice Strain	Crystalline Size (nm)	Cell Volume (Å^3)	Bond Length (Å)			R_{wp} (%)	GoF (x^2)
								a (Å)	b (Å)	c (Å)				Sr-O	Ti-O	Zr-O		
SZGG8R	0.0	100.0	100.0	200	7.5	$SrZrO_3$	Orthorhombic	5.8049 (28)	5.8071 (10)	8.2125 (1)	0.60 (0.01)	816.4 (4.3)	276.84 (0.01)	2.6890	-	1.9130	4.498	4.31
SZGG2RR	10.0	90.0	100.0	200	7.5	$SrZr_{0.9}Ti_{0.1}O_3$	Cubic	4.0907 (1)	-	-	0.50 (0.005)	826.3 (9.7)	68.83 (0.03)	2.8918	2.0448	2.0448	3.867	3.59
SZGG3R	20.0	80.0	100.0	200	7.5	$SrZr_{0.8}Ti_{0.2}O_3$	Cubic	4.0737 (10)	-	-	0.47 (0.005)	812.4 (3.2)	68.14 (0.01)	2.8881	2.0422	2.0422	3.984	3.58
SZGG4R	30.0	70.0	100.0	200	7.5	$SrZr_{0.74}Ti_{0.26}O_3$	Cubic	4.0505 (16)	-	-	0.48 (0.01)	670.6 (7.1)	66.93 (0.01)	2.8852	2.0413	2.0413	4.448	3.81
SZGG1RR	50.0	50.0	100.0	200	7.5	$SrZr_{0.527}Ti_{0.473}O_3$	Cubic	3.9978 (6)	-	-	0.44 (0.004)	515.2 (6.1)	64.69 (0.03)	2.8269	1.9991	1.9991	7.935	5.82
SZGG5R	70.0	30.0	100.0	200	7.5	$SrTi_{0.67}Zr_{0.33}O_3$	Cubic	3.9584 (5)	-	-	0.43 (0.005)	558.9 (1.5)	62.02 (0.02)	2.7989	1.9792	1.9792	4.628	3.76
SZGG16R	80.0	20.0	100.0	200	7.5	$SrTi_{0.8}Zr_{0.2}O_3$	Cubic	3.9382 (5)	-	-	0.22 (0.003)	588.5 (0.7)	61.07 (0.07)	2.7848	1.9691	1.9691	4.485	3.69
SZGG6R	90.0	10.0	100.0	200	7.5	$SrTi_{0.9}Zr_{0.1}O_3$	Cubic	3.9275 (5)	-	-	0.08 (0.005)	599.9 (5.7)	60.58 (0.02)	2.7771	1.9637	1.9637	6.163	4.32
SZGG7R	100.0	0.0	100.0	200	7.5	$SrTiO_3$	Cubic	3.9133 (6)	-	-	0.01 (0.009)	469.0 (6.8)	59.92 (0.02)	2.7612	1.9525	-	6.423	4.912
SZGG15R	50.0	50.0	100.0	125	7.5	$SrTi_{0.5}Zr_{0.5}O_3$	Cubic	3.9868 (2)	-	-	0.20 (0.01)	334.9 (9.6)	63.39 (0.01)	2.8194	1.9936	1.9936	3.65	3.28
SZGG14R	50.0	50.0	100.0	150	7.5	$SrTi_{0.5}Zr_{0.5}O_3$	Cubic	3.9873 (1)	-	-	0.24 (0.01)	342.1 (4.2)	63.37 (0.01)	2.8191	1.9934	1.9934	4.23	3.60
SZGG13R	50.0	50.0	100.0	175	7.5	$SrTi_{0.5}Zr_{0.5}O_3$	Cubic	3.9884 (2)	-	-	0.35 (0.01)	429.1 (8.2)	63.44 (0.01)	2.8202	19942	1.9942	4.02	3.59
SZGG10	50.0	50.0	100.0	200	10	$SrTi_{0.5}Zr_{0.5}O_3$	Cubic	3.9990 (5)	-	-	0.62 (0.01)	408.2 (4.2)	63.48 (0.02)	2.8207	1.9945	1.9945	12.724	8.83
SZGG11R	50.0	50.0	100.0	200	12.5	$SrTi_{0.501}Zr_{0.499}O_3$	Cubic	4.0000 (3)	-	-	0.82 (0.01)	454.9 (7.8)	64.01 (0.01)	2.8296	2.0024	2.0024	6.142	5.10
SZGG12R	50.0	50.0	100.0	200	15	$SrTi_{0.5}Zr_{0.5}O_3$	Cubic	4.0014 (4)	-	-	0.88 (0.01)	217.6 (9.2)	64.06 (0.02)	2.8363	2.0015	2.0015	5.276	3.35

Note: *Pm3m* = Crystalline structure orthorhombic.
Pbnm = Crystalline structure cubic.
[a] The lattice parameters "a" were only determined for solid solution $SrTi_{1-x}Zr_xO_3$, and "a", "b" and "c" were only determined for $SrZrO_3$; these values were calculated considering anisotropic lattice strain, particle size and preferential orientation as parameters for the Rietveld refinement analyses.

S3. Microstructural features of the SS particles prepared under alkaline hydrothermal conditions with vigorous fluid stirring

Fig. S2 shows the morphology of particles obtained at 200 ºC for 6 h in a 5 M KOH solution with different coprecipitated gel compositions. Generally, the micrographs showed marked differences in crystal particle size above Ti^{4+} contents of 10 mol%. In all cases, the microstructural results indicated that secondary phases were not formed simultaneously during the crystallisation of the perovskite-structured cubic (SS1) and orthorhombic Zr^{4+}-rich (SS2) crystals. Furthermore, the particles' morphology is irrespective of the crystalline phase formed (Figs. S2a–S2f); the particles exhibited a cubic habit and a pseudocuboidal shape, consistent with the morphological crystalline features of the perovskite species, i.e. $CaTiO_3$. The particles corresponding to the new cubic-structured SS1s were visible on the FE-SEM micrographs of powder samples prepared with Ti^{4+} contents over 10.0 mol% (Figs. S3a–S3f). Therefore, the mesocrystals SS1 formation was difficult to determine on the samples prepared with Ti^{4+} contents between 10.0 and 90.0 mol %. Based on the particle microstructural aspect, we argue that the growth of the pseudocuboidal-shaped particles occurred preferentially along the {100} planes, and that the epitaxial mechanism enhanced their coarsening in the {100} plane direction. Additionally, other specific crystalline aspects of the cubic- and orthorhombic-structured samples were revealed by TEM and SAED images. The typical images shown in Figure S3 indicate that each hydrothermally crystallised particle is, in nature, a single crystal. The SAED pattern confirmed this feature on all the particles; however, a marked difference in the pattern orientation between the single-phases ($SrZrO_3$ and $SrTiO_3$) and the SS1 particles, as seen in the inset micrographs in Figure S3. Interestingly, the SADE patterns in the samples with a partial substitution of 30.0 and 70.0 mol% Ti^{4+}, exhibit similar plane distribution, indicating that their crystalline structure belongs to the cubic geometry. The preferential orientation is likely due to crystal growth that proceeds perpendicular to the zone axis (001). Furthermore, the plane dots distribution does not exhibit additional dots corresponding to a superlattice associated with the tetragonal symmetry. Therefore, the crystalline features revealed on the SSs1 confirm that the crystallised powders with intermediate compositions in the $SrTiO_3$–$SrZrO_3$ belong to the cubic crystalline structure with space group *Pm3m*.

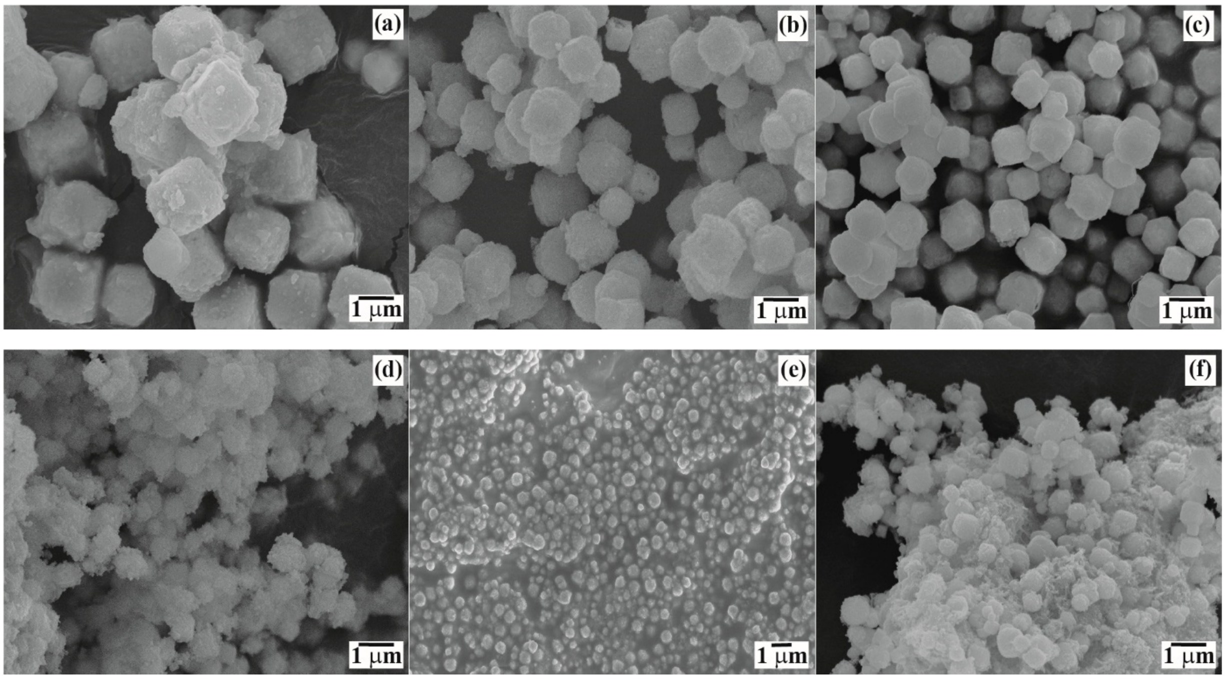

Figure S2. FE-SEM micrographs of particles of the solid solutions $SrTi_{1-x}Zr_xO_3$ and $SrZr_{1-x}Ti_xO_3$, obtained under hydrothermal conditions at 200 °C for 6 h, using a 5 M KOH and cationic solutions with equimolar volume of 7.5 ml, with different contents of Ti^{4+} a) 90.0, (b) 80.0, (c) 70.0, (d) 30.0, (e) 20.0, (f) 10.0 %mol.

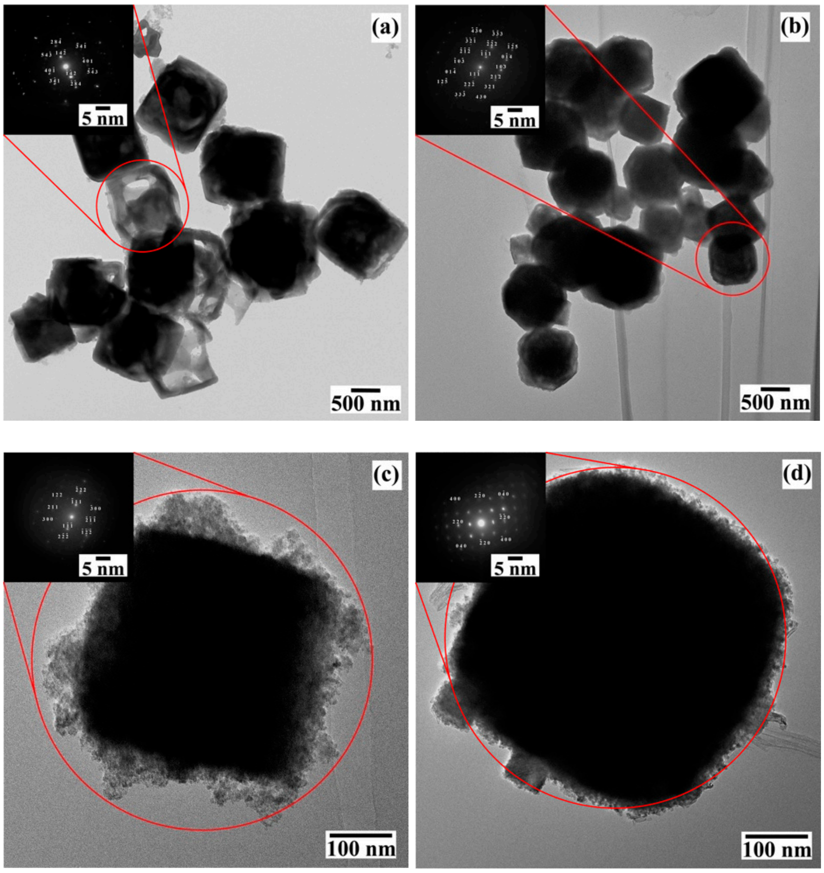

Figure S3. TEM micrographs of powders corresponding to the SSs $SrTi_{1-x}Zr_xO_3$ and $SrZr_{1-x}Ti_xO_3$, obtained under hydrothermal conditions at 200 °C for 6 h, using a 5 M KOH and cationic solutions with equimolar volume of 7.5 ml, with different contents of Ti^{4+} a) 0.0, (b) 30.0, (c) 70.0, (d) 100.0 mol%.

S4. Optical characterisation of cubic and orthorhombic SS particles prepared by coprecipitation and hydrothermal processing

The bandgap energies of the cubic and orthorhombic perovskite powders were calculated from ultraviolet spectral reflectance (300–700 nm) data. The Kubelka-Munk function (Eq. S5) was used to determine the plot shown in Fig. S4. Figure 4 portrays the direct band gap plot of the reaction products prepared at 200 °C for 6 h with a 5 M KOH solution, varying the Ti^{4+} content in the perovskite cubic $SrTi_{1-x}Zr_xO_3$ and $SrZr_{1-x}Ti_xO_3$ solid solutions. These results indicated that the systematic direct band gap energies of single-phase ($SrTiO_3$, $SrZrO_3$) and intermediate SS1 samples varied within a narrow range of 3.12 to 3.57 eV, confirming the perovskite-semiconductor nature of the compounds prepared within the binary $SrTiO_3$–$SrZrO_3$ system. The average direct bandgap of 3.12 eV also has a minor statistical standard deviation of ±0.03 eV.

Kubelka–Munk equation, i.e.,

$$F(R) = \frac{1-R^2}{2R} \qquad \text{Eq. S5}$$

where R is the observed reflectance of the samples.

The NIR Solar reflectance (R*) measurements were carried out in accordance with ASTM standard G173–03. The solar reflectance of the developed pigments was calculated over the wavelength range of 700–2500 nm using the Eq. S2:

$$R^* = \frac{\int_{700}^{2500} r(\lambda)i(\lambda)d(\lambda)}{\int_{700}^{2500} i(\lambda)d(\lambda)} \qquad \text{Eq. S2}$$

where r(λ) is the spectral reflectance experimentally obtained, and i(λ) is the solar spectral irradiance ($Wm^{-2}mm^{-1}$) obtained from ASTM standard number G173–03.

Table S4 summarises the optical parameters associated with the hydrothermally prepared cubic perovskite powders. The values of the crystal size correspond to the pseudocuboidal-shaped particles calculated from the linear measurements carried out on the basal surface of the particles along their middle axes. Furthermore, the CIELab coordinates and other colour data were derived from the analysis of the UV-vis spectra of each powder. These values are consistent, with slight differences attributable to Ti^{4+} partially substituting for Zr^{4+} in the octahedral sites of the chemically stable cubic perovskite. Table S4 also includes the solar irradiance value (%R*) for comparison, which is used as a standard for the design of white solar cells with high solar irradiance performance.

It is worth emphasising that the perovskite cubic powders exhibit high values comparable to those of TiO_2; thus, they can be considered for potential solar irradiance applications.

Table S4. Summary of the particle sizes, bandgap energy, solar reflectance, CIEL*a*b* colour parameters, chroma, RGB values, and observed colour hue $SrZr_{1-x}Ti_xO_3$ and $SrTi_{1-x}Zr_xO_3$ powders synthesised under hydrothermal conditions with 5 M KOH solution.

Sample ID	Ti (% at)	Zr (% at)	Sr (% at)	Temperature (°C)	Solar irradiance (%R*)	Bandgap (eV)	Crystal Size (nm)	CIELab-Coordinates			RGB Colour Coordinates			Chroma Cab*	Colour Hue
								L*	a*	b*	R	G	B		
SZGG8R	0.0	100.0	100.0	200	79.85	3.57	770.5 (10.2)	92.20	-1.15	1.13	231	233	230	1.61	
SZGG2R	10.0	90.0	100.0	200	80.94	3.54	980.1 (218)	95.08	-0.97	0.67	239	241	239	1.18	
SZGG3R	20.0	80.0	100.0	200	79.54	3.51	763.5 (15.6)	92.13	-1.23	1.22	230	233	229	1.73	
SZGG4	26.0	74.0	100.0	200	86.49	3.49	691.0 (21.9)	96.23	-0.62	0.55	243	244	243	0.83	
SZGG1RR	47.2	52.7	100.0	200	78.22	3.45	490.1 (10.3)	91.50	-1.14	-1.14	232	234	231	1.61	
SZGG5R	67.0	33.0	100.0	200	78.39	3.39	550.9 (10.5)	91.42	-1.12	-1.14	227	231	232	1.59	
SZGG16R	80.0	20.0	100.0	200	77.60	3.30	467.9 (19.7)	91.32	-1.13	1.15	231	234	230	1.61	
SZGG6	90.0	10.0	100.0	200	81.20	3.21	453.8 (21.5)	95.20	-0.77	1.08	241	242	239	1.33	
SZGG7R	100.0	0.0	100.0	200	79.80	3.12	450.6 (11.1)	92.35	-0.99	0.97	231	234	231	1.38	
SZGG15R	50.0	50.0	100.0	125	81.04	3.46	299.8 (13.5)	92.79	-0.84	0.92	233	235	232	1.24	
SZGG14R	50.0	50.0	100.0	150	80.03	3.44	308.4 (17.2)	92.24	-0.85	0.84	231	233	231	1.19	
SZGG13R	50.0	50.0	100.0	175	79.58	3.46	312.6 (9.8)	92.01	-0.87	0.94	231	233	230	1.28	
TiO_2 (Rutile)	-	-	-	-	79.01	-	5000	97.85	0.32	1.65	251	249	246	1.68	

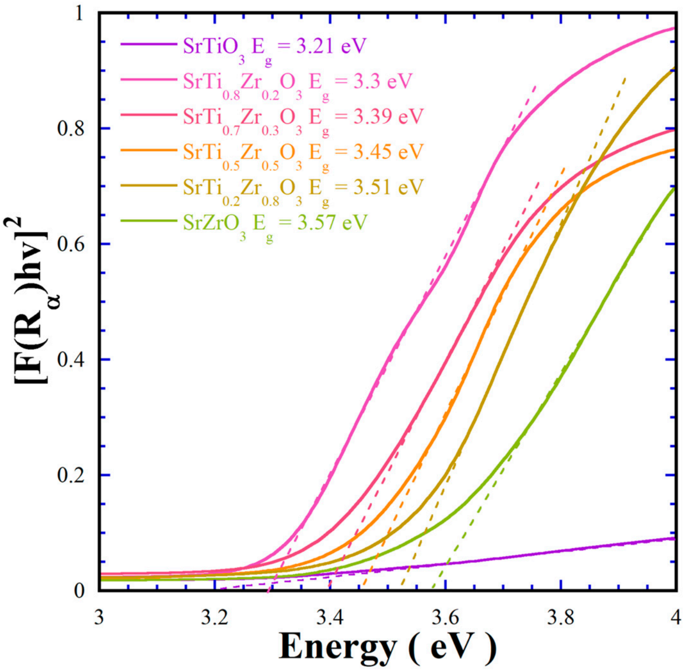

Figure S4. Kubelka-Munk curves of perovskite powders prepared with various contents of Ti^{4+} under hydrothermal conditions at 200 °C for 6 h with a 5 M KOH solution stirred at 130 r.p.m.
